# Identification of Cyclic Sulfonamides with an *N-*Arylacetamide Group as α-Glucosidase and α-Amylase Inhibitors: Biological Evaluation and Molecular Modeling

**DOI:** 10.3390/ph15010106

**Published:** 2022-01-17

**Authors:** Furqan Ahmad Saddique, Matloob Ahmad, Usman Ali Ashfaq, Muhammad Muddassar, Sadia Sultan, Magdi E. A. Zaki

**Affiliations:** 1 Department of Chemistry, Government College University, Faisalabad 38000, Pakistan; furqanas123@gmail.com; 2 Department of Bioinformatics and Biotechnology, Government College University, Faisalabad 38000, Pakistan; ashfaqua@gcuf.edu.pk; 3 Department of Biosciences, COMSATS University Islamabad, Park Road, Islamabad 45500, Pakistan; mmuddassar@comsats.edu.pk; 4 Faculty of Pharmacy, Puncak Alam Campus, Universiti Teknologi MARA, Bandar Puncak Alam 42300, Selangor Darul Ehsan, Malaysia; drsadia@uitm.edu.my; 5 Atta-ur-Rahman Institute for Natural Products Discovery (AuRIns), Puncak Alam Campus, Universiti Teknologi MARA, Bandar Puncak Alam 42300, Selangor Darul Ehsan, Malaysia; 6 Department of Chemistry, Faculty of Science, Imam Mohammad Ibn Saud Islamic University (IMSIU), Riyadh 11623, Saudi Arabia

**Keywords:** 1,2-benzothiazine, acetamide, synthesis, α-glucosidase, α-amylase, molecular modeling, anti-diabetic

## Abstract

Diabetes mellitus (DM), a complicated metabolic disorder, is due to insensitivity to insulin function or reduction in insulin secretion, which results in postprandial hyperglycemia. α-Glucosidase inhibitors (AGIs) and α-amylase inhibitors (AAIs) block the function of digestive enzymes, which delays the carbohydrate hydrolysis process and ultimately helps to control the postprandial hyperglycemia. Diversified 2-(3-(3-methoxybenzoyl)-4-hydroxy-1,1-dioxido-2*H*-benzo[*e*][1,2]thiazin-2-yl)-*N*-arylacetamides were synthesized and evaluated for their in vitro inhibitory potential against α-glucosidase and α-amylase enzymes. The compounds with chloro, bromo and methyl substituents demonstrated good inhibition of α-glucosidase enzymes having IC_50_ values in the range of 25.88–46.25 μM, which are less than the standard drug, acarbose (IC_50_ = 58.8 μM). Similarly, some derivatives having chloro, bromo and nitro substituents were observed potent inhibitors of α-amylase enzyme, with IC_50_ values of 7.52 to 15.06 μM, lower than acarbose (IC_50_ = 17.0 μM). In addition, the most potent compound, *N*-(4-bromophenyl)-2-(4-hydroxy-3-(3-methoxybenzoyl)-1,1-dioxido-2*H*-benzo[e][1,2]thiazin-2-yl)acetamide (**12i**), was found to be a non-competitive and competitive inhibitor of α-glucosidase and α-amylase enzymes, respectively, during kinetic studies. The molecular docking studies provided the binding modes of active compounds and the molecular dynamics simulation studies of compound **12i** in complex with α-amylase also showed that the compound is binding in a fashion similar to that predicted by molecular docking studies.

## 1. Introduction

Diabetes mellitus (DM) is one of the most stressful, chronic and recurrent diseases, affecting approximately 0.5 billion humans word over [1]. In DM, glucose is accumulated in the blood and ultimately results in hyperglycemia [2]. Every year, approximately five million deaths are attributed to DM. In addition, it also increases the chances of thrombosis, heart illness, and microvascular complications like blindness, kidney failure, and supplemental neuropathy [3]. The beta cells destruction due to heredity problems or auto-immune destruction can cause complete insulin inadequacy, and this condition is known as type 1 DM (or T1DM) or insulin-dependent DM [4]. People affected by this stage of the disease are likely to inject artificial insulin and may have insulin-resistant conditions in their life history that often lead to type 2 diabetes [5]. In the most common form of DM, known as insulin-independent type 2 DM, or T2DM, the body fails to incorporate insulin properly, which ultimately results in insulin resistance. T2DM may occur at any stage of life. Approximately 60–90% of T2DM patients may have obesity because they become glucose tolerant [6].

The requirement of most of the body’s energy is fulfilled via the consumption of starch. The most beneficial enzymes, which degrade starch for its usefulness, are α-amylases (salivary and pancreatic) and α-glucosidases (small intestinal) [7]. α-Amylase (α-1,4 glucan-4-glucanohydrolase) initially breaks the starch into oligosaccharides by breaking the α-1,4-glucan bonds. Thus, the first step in the utilization of carbohydrates is the formation of oligosaccharides from polysaccharides catalyzed by α-amylase enzymes. Disaccharides and oligosaccharides (the un-absorbable carbohydrates) then interact with the α-glucosidase, an enzyme found in the epithelial mucosa of the small intestine. α-Glucosidase enzymes cleave the un-absorbable carbohydrates into simple and intestinally absorbable carbohydrates, which are then diffused into the blood stream and ultimately result in an increased blood sugar level [8]. Diet and exercise are the first steps to treat T2DM. But if this practice fails to control the glucose level in the blood, drug treatment is recommended. Medical treatment focuses on the lowering of postprandial glucose levels in the blood to control the progression of diabetes [9]. A general consideration to control the blood glucose level is the inhibition of carbohydrate hydrolyzing enzymes (α-amylase and α-glucosidase) using various natural and synthetic compounds [10,11,12,13].

The use of inhibitors of carbohydrate hydrolyzing enzymes may be an effective alternative as a first-line treatment for T2DM, as these target the postprandial hyperglycemia, which may be an independent risk factor for heart problems [14]. However, the presently used α-glucosidase inhibitors (AGIs) show some adverse side effects, like diarrhea, flatulence, hypoglycemia, stomach ache, drug resistance, damage of the liver, weight gain and heart diseases [15,16,17,18,19,20]. The effective α-glucosidase inhibitors, like miglitol, acarbose and voglibose, are most commonly employed, but these have failed to inhibit the α-amylase activity remarkably. An ideal template could competitively block the activity of both α-amylase and α-glucosidase and thus effectively control T2DM [21].

**Figure 1:**
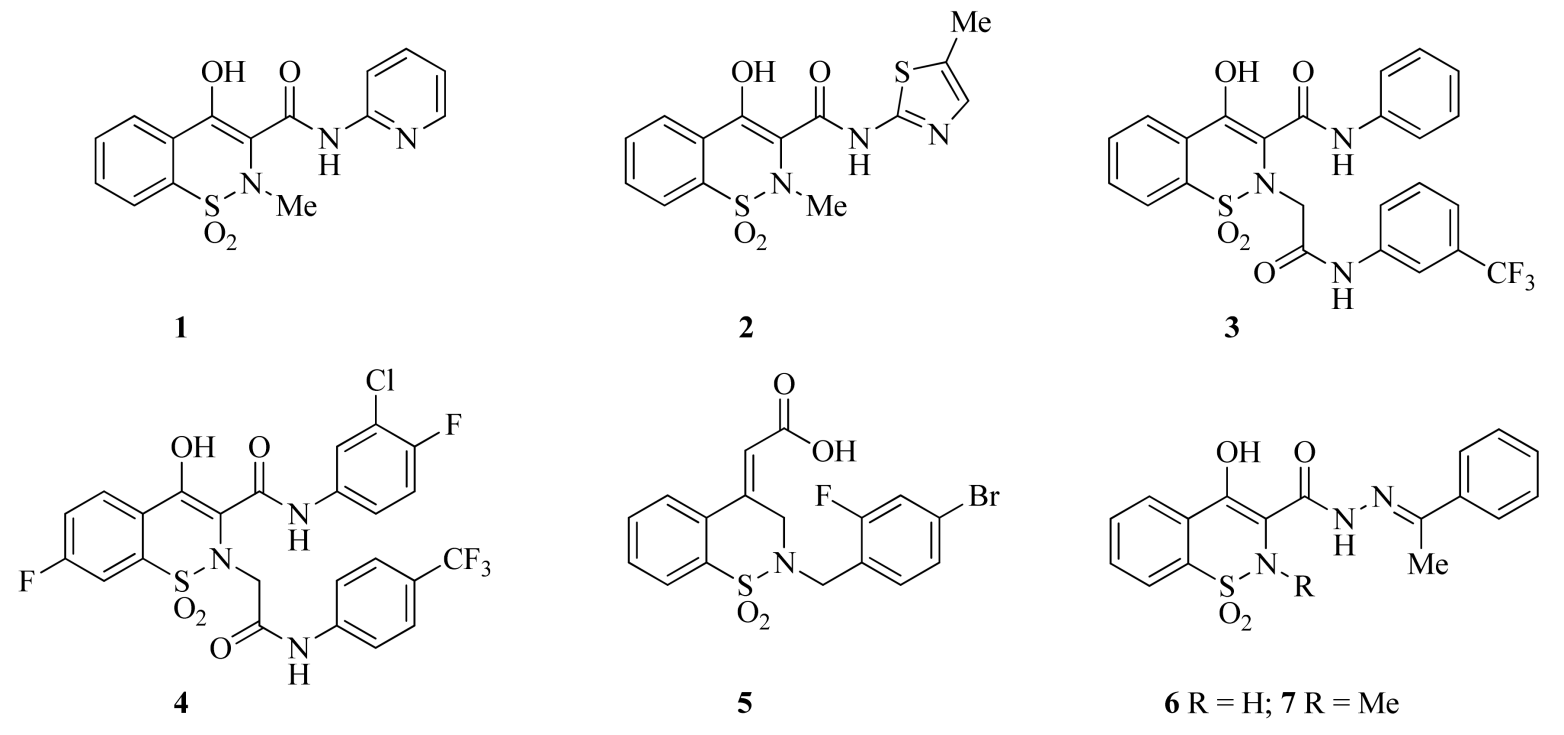
Biologically potent 1,2-benzothiazine 1,1-dioxides. Compound **1** and **2** (antiinflammatory agents), **3** (11*β*-HSD1 inhibitor), **4** (SARS-CoV-2 inhibitor), **5** (aldose reductase enzyme inhibitor), **6** and **7** (α-glucosidase inhibitors).

Benzothiazine 1,1-dioxides have prevailed in heterocyclic chemistry because of their enormous applications in different fields of science. In particular, 1,2-benzothiazine 1,1-dioxide carboxamides (oxicams) have dominated the 1,2-benzothiazine 1,1-dioxides chemistry. Piroxicam **1** (Figure 1), the most potent oxicam, is employed to cure osteoarthritis, rheumatoid arthritis and other inflammations [22], while meloxicam **2** (Figure 1) is effective at lower concentrations and possesses COX-2/COX-1 selectivity [23,24]. It results in few gastrointestinal disorders and controls pain and stiffness in clients [25]. After the discovery of these biologically potent scaffolds, numerous 1,2-benzothiazine 1,1-dioxides were synthesized and reported to have significant pharmacological activities. For example, 1,2-benzothiazine-*N*-arylacetamides **3** and **4** (Figure 1) were found to be good inhibitors of human 11*β*-hydroxysteroid dehydrogenase type 1 (11*β*-HSD1) and severe acute respiratory syndrome coronavirus 2 (SARS-CoV-2), with IC_50_ values of 0.041 and 0.88 μM, respectively [26,27]. Similarly, the derivative **5** (Figure 1) exhibited excellent inhibition of aldose reductase enzymes, with an IC_50_ value of 0.11 μM [28]. Furthermore, the 1,2-benzothiazine-3-carbohydrazides **6** and **7** (Figure 1) were proven good α-glucosidase inhibitors, with IC_50_ values of 3.9 and 4.2 μM, respectively [29,30]. Some other biological activities shown by 1,2-benzothiazine derivatives are MAO inhibitors [31], α-glucosidase inhibitors [32,33], anti-inflammatory agents [34], antiviral agents [35,36,37,38], anticancer agents [39,40], antitumor agents [41], cholinesterase inhibitors [42] and anti-microbial agents [43]. In addition, various sulfonamides were also found to be good inhibitors of α-glucosidase [44] and α-amylase [45] enzymes. We synthesized a library of 1,2-benzothiazine-*N*-arylacetamides and screened them for their in silico and in vitro inhibitory potential against α-glucosidase and α-amylase enzymes.

## 2. Results and Discussion

### 2.1. Synthetic Chemistry

**Scheme 1:**
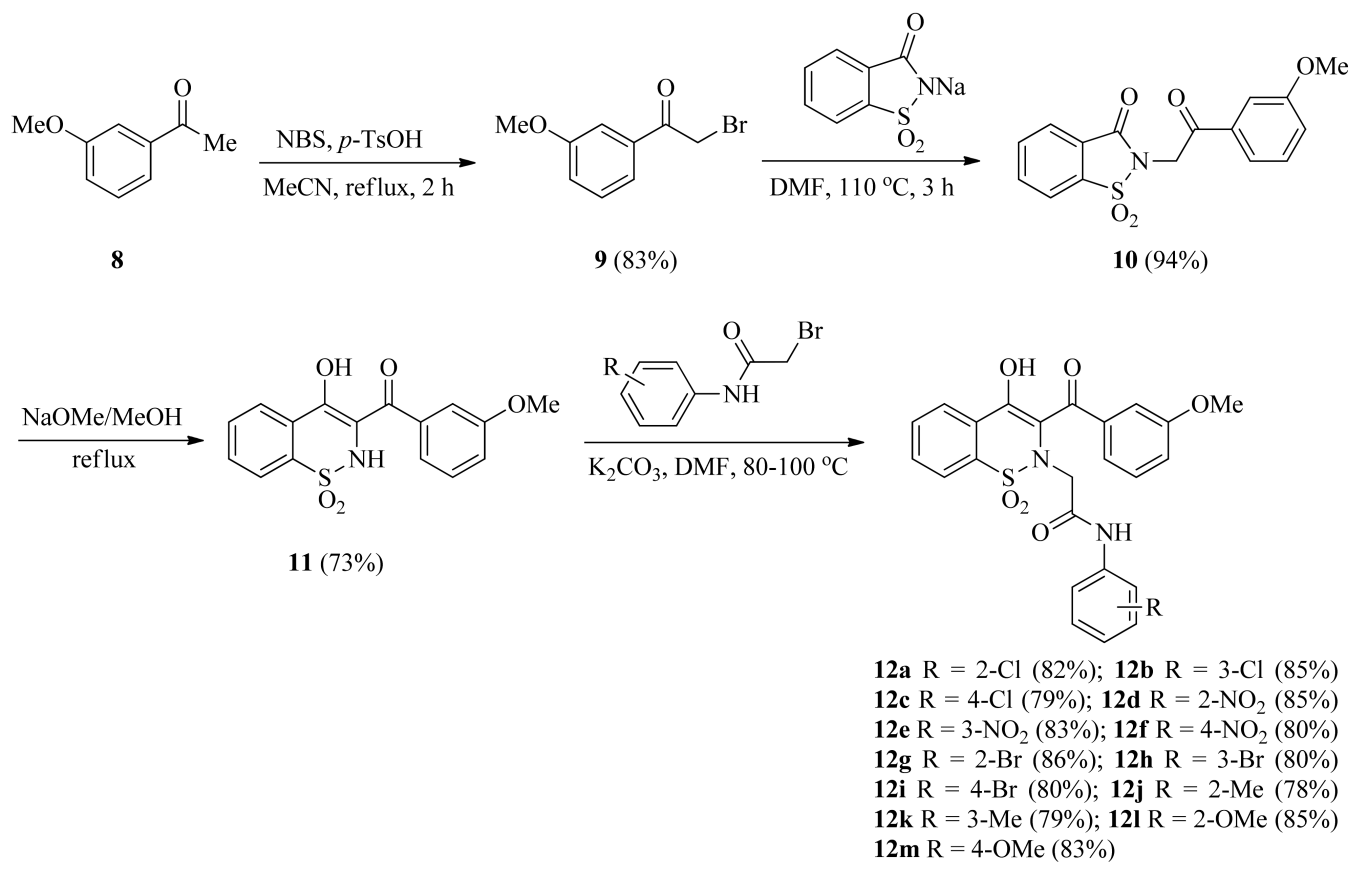
Synthetic route to 2-(3-(3-methoxybenzoyl)-4-hydroxy-1,1-dioxido-2*H*-benzo[e][1,2]thiazin-2-yl)-*N*-arylacetamides **12a–m**.

The 3-(3-methoxybenzoyl)-1,2-benzothazine **11** was synthesized according to the reported method [46,47], starting from the α-bromination of 3-methoxyacetophenone **8** with *N*-bromosuccinamide (NBS) [48] followed by a substitution reaction with sodium saccharin to get bezoisothiazolone **10**. The base catalyzed Gabriel-Colman rearrangement [35,49] converted the bezoisothiazolone **10** into isomeric 3-(3-methoxybenzoyl)-1,2-benzothazine **11,** which was coupled with a variety of 2-bromo-*N*-arylacetamides [50] under basic conditions (K_2_CO_3_) in DMF. Dilution with water and then addition of cold dil. HCl (20%) furnished the requisite 2-(3-(3-methoxybenzoyl)-4-hydroxy-1,1-dioxido-2*H*-benzo[e][1,2]thiazin-2-yl)-*N*-arylacetamides **12a–m** in good yields (78–86%) (Scheme 1). Reactions were also carried out for longer reaction time (beyond 2–2.5 h) but failed to enhance the product yields. Furthermore, reactions in acetonitrile also provided similar product yields but in 4–4.5 h.

### 2.2. Spectroscopic Characterization

^1^H NMR, ^13^C NMR and HRMS (ESI) spectroscopic techniques were used for the structural characterization of synthesized compounds. Signals at 3.95–4.20 ppm in the ^1^H NMR spectra confirmed the presence of methylene (CH_2_) protons. Similar observations have also been reported in literature for the structurally related compounds [26,32,49]. Singlets at 3.64–3.86 and 1.96–2.17 ppm were assigned to the OCH_3_ (methoxy) and CH_3_ (methyl) protons, respectively. Similarly, singlets at 9.16–10.48 and 14.92–15.01 ppm were attributed to the NH and OH protons, respectively, as reported in the literature [26,32,51,52]. However, peaks for the OH proton in most of the spectra were not observed, which is consistent with the previous observations [26,32]. The aromatic protons appeared in the range of 6.50–8.50 ppm. The observed molecular ion peaks in the HRMS (ESI) spectra were found in correspondence to the calculated values.

### 2.3. In Silico Analysis

#### 2.3.1. Molecular Docking

The target of every synthetic work is to find the biologically effective candidates. However, synthesis of bulk of organic compounds and their biological screening is a time-consuming and costly work, which can be avoided with the application of an in silico approach [53,54,55,56]. Molecular docking is a well-known and versatile in silico technique that helps to target the biologically effective templates among the libraries of compounds before their synthesis. It predicts interaction modes between the optimized conformers of different ligands and the receptor protein, which helps to specify the synthetic targets [57,58,59]. Keeping in view the potential of this tool, we elaborated the detailed molecular docking studies of synthesized 1,2-benzothiazine-*N*-arylacetamides with the help of Molecular Orbital Environment (MOE) software to explore the binding modes between the ligands and the targeted enzymes (α-glucosidase and α-amylase). Compounds were graded on the basis of root mean square deviations (RMSD), bindings with the reference residues and docking scores (Table 1) [60].

##### α-Glucosidase (Molecular Docking)

**Figure 2:**
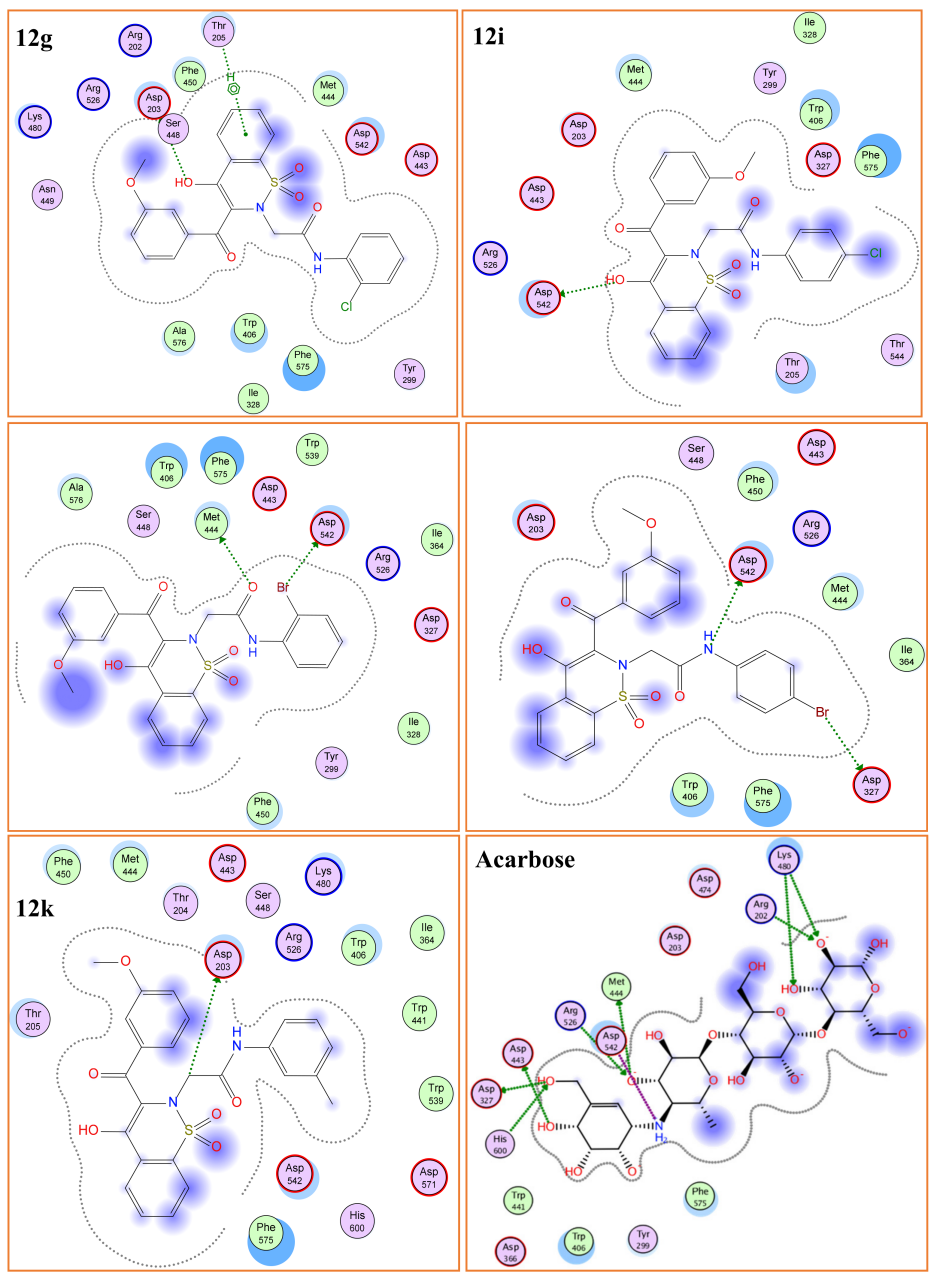
Binding interactions (2D maps) of potent compounds (**12a**, **12c**, **12g**, **12i**, **12k**) and the reference drug, acarbose, against α-glucosidase enzyme. Ligand exposure points are indicated with blue color, while the binding interactions of ligands with the receptor enzyme are indicated with green dotted lines.

The optimized structures of potent derivatives were docked into the selective pocket of the α-glucosidase enzyme (N-terminal maltase glucoamylase (PDB ID: 2QMJ)) [16,61], and it was observed that most of the derivatives showed good docking scores and binding interactions with the selective residues (Asp327, Asp203, Arg526, Asp542, His600) (Table 1, Figure 2). The ligands **12a**, **12c**, **12g**, **12i** and **12k**, having docking scores from −12.50 to −13.30 Kcal/mol with the RMSD values less than 2 Å, were ranked as the most effective α-glucosidase inhibitors. The docking scores of these derivatives were found closer to the docking score of acarbose (−16.18 Kcal/mol). Two-dimensional interaction modes conveyed that most of the derivatives showed interactions with Asp327, Asp542 and Asp203 among the concerned residues and were found important regarding the inhibition of targeted enzyme (Figure 2, Table 1). The salacinol and its derivatives also blocked the α-glucosidase enzyme via interacting with Asp327, Asp542 and Asp203 residues during in silico screening [62]. In a recent report, the anthocyanins and anthocyanidins were proven good inhibitors of α-glucosidase enzyme, as these scaffolds showed bindings with Asp203, Asp327 and Asp542 residues [63]. Similarly, aglycone of curculigoside A and derivatives also showed interactions with these three residues during molecular docking studies against the α-glucosidase enzyme [64].

The ligand **12a** showed two interactions: a hydrogen bond between enolic-OH and residue Asp203 and an arene-H interaction with Thr205 residue. These interactions and low RMSD value (1.28 Å) justify the low binding energy (−12.50 Kcal/mol) of the ligand **12a**. The ligand **12c**, having an RMSD value of 1.29 Å and binding energy of −12.80 Kcal/mol, formed a hydrogen bond with Asp542 via its OH group. Similarly, the compound **12g** exhibited two interactions: a dipole–dipole interaction with Asp542 using its bromo group and a hydrogen bond with Met444 using oxygen of the CONH group. These interactions justify the low binding energy (−12.98 Kcal/mol) and RMSD value (1.22 Å) of ligand **12g**. The most effective results were shown by the compound **12i** among the series, which showed interactions with the residues Asp327 and Asp542. The Asp327 interacted with the bromo atom, while Asp542 formed a hydrogen bond with the NH group of this ligand. Furthermore, the best docking score (−13.30 Kcal/mol) and the lowest RMSD value (1.01 Å) were witnessed for this ligand. Another ligand, **12k**, exhibited a good docking score (−13.22 Kcal/mol) and a low RMSD value of 1.12 Å. This ligand formed an interaction with the residue Asp203 via its methylene moiety. Hence, the ligands **12a**, **12c**, **12g**, **12i** and **12k** were observed as good inhibitors of α-glucosidase enzyme, having good binding modes, binding energies and RMSD values. Acarbose was also redocked into the receptor pocket to check the validity of the experiment and showed a good docking score with significant bindings with the concerned residues (Table 1, Figure 2).

##### α-Amylase (Molecular Docking)

**Figure 3:**
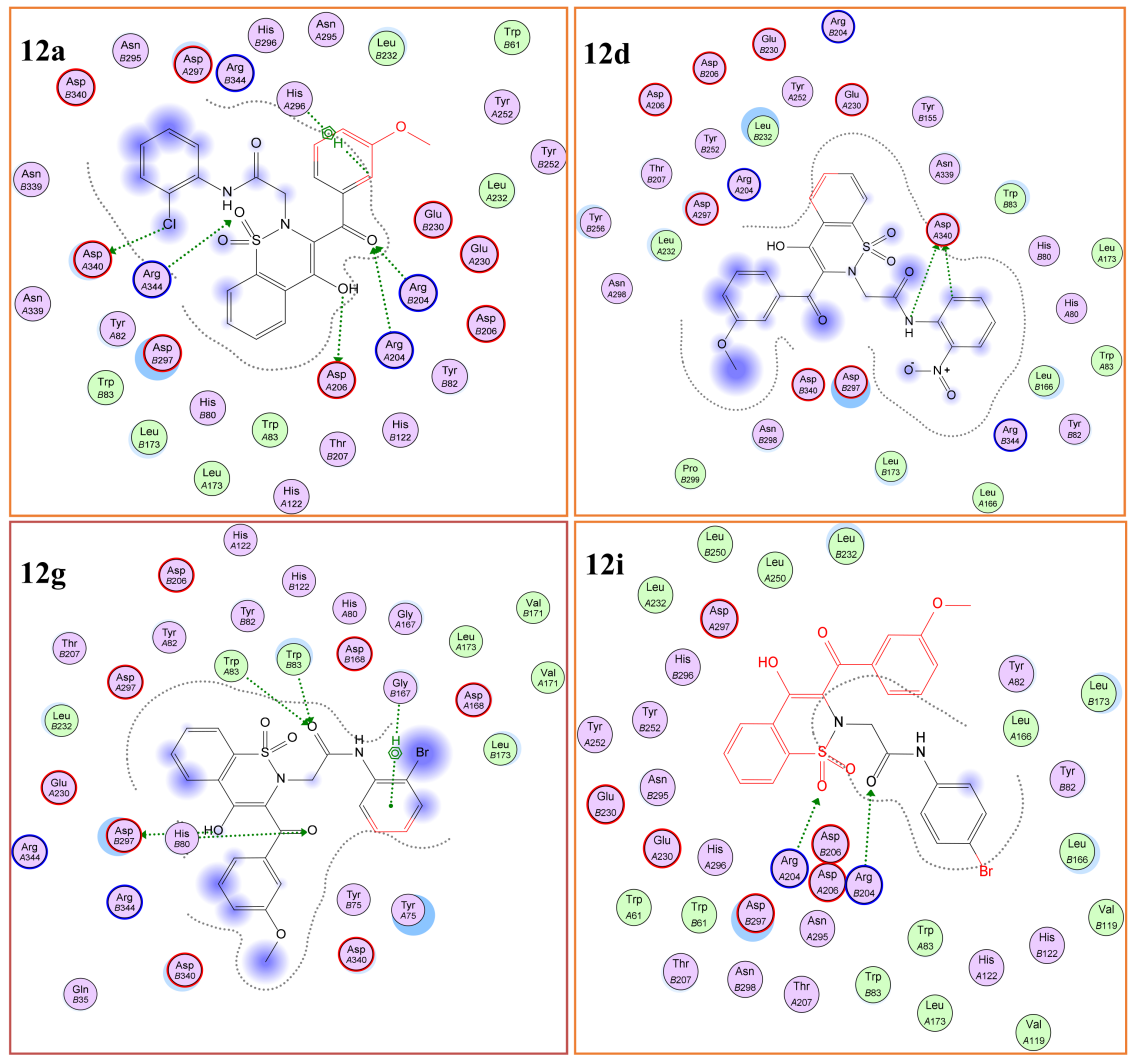

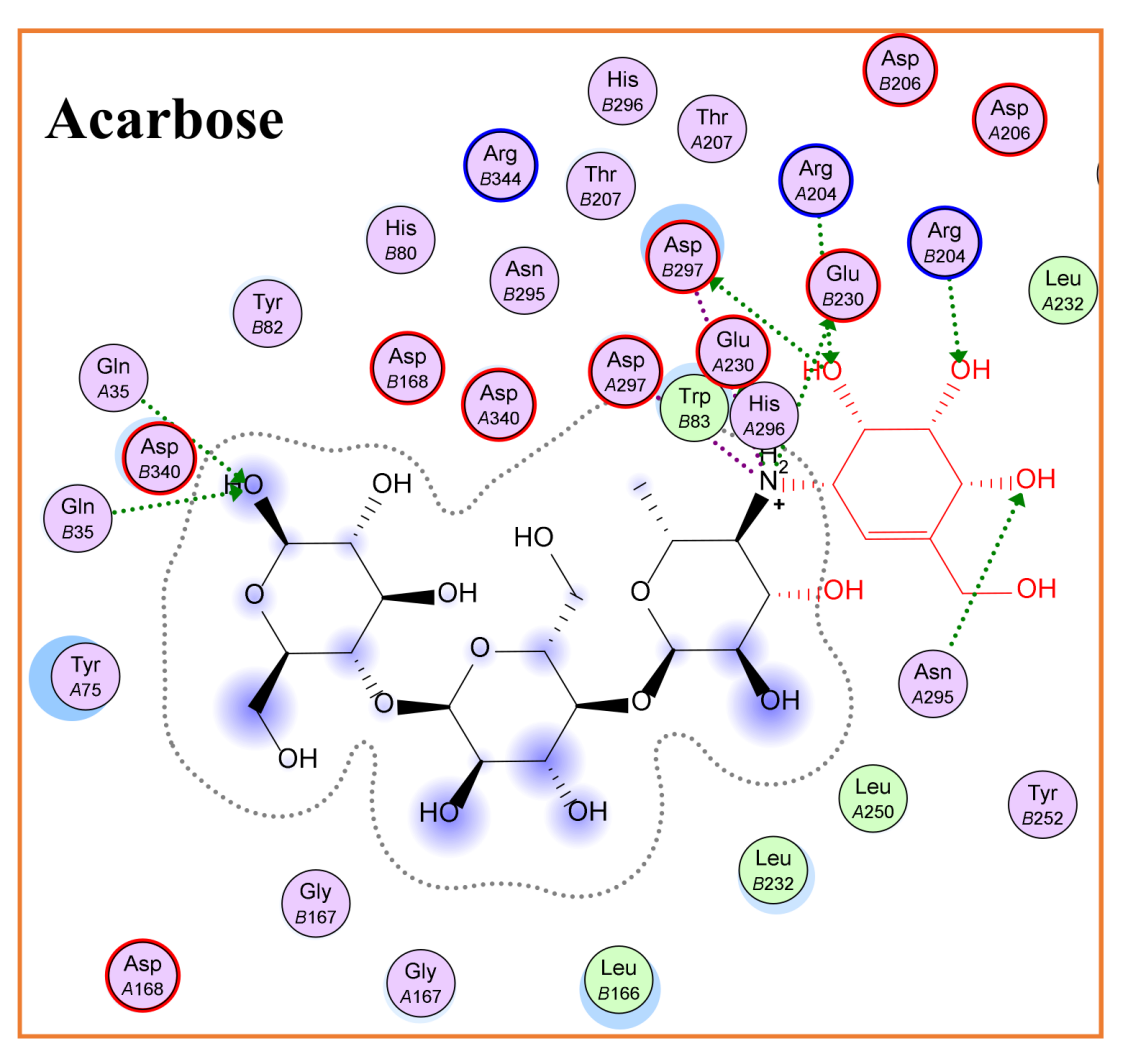
Binding interactions (2D maps) of potent compounds (**12a**, **12d**, **12g**, **12i**) and the reference drug, acarbose, against α-amylase enzyme. Ligand exposure points are indicated with blue color, while the binding interactions of ligands with the receptor enzyme are indicated with green dotted lines.

The molecular docking studies of the 1,2-benzothiazine derivatives against the Aspergillus oryzae α-amylase (PDB ID: 7TAA) [65,66] were found effective, as some of the derivatives showed the best docking scores (−14.60 to −16.13 Kcal/mol), which were comparable to the docking score of the standard drug, acarbose (−17.69 Kcal/mol) (Table 1). In addition, these scaffolds also demonstrated excellent binding interactions with the selected residues (Trp83, Asp340, Arg344, Arg204, Glu230, Lys209) (Figure 3) and low RMSD values (Table 1). Among the screened derivatives, the ligands **12a**, **12d**, **12f**, **12g** and **12i** exhibited the most promising in silico inhibitions of α-amylase enzymes, as indicated by their low docking score, low RMSD values and favorable interactions with the selected residues. These ligands were found to have −15.24, −14.89, −14.60, −15.89 and −16.13 Kcal/mol docking scores, respectively. Furthermore, the RMSD values of these ligands were also found to be less than 2 Å (Table 1). 

A look at the two-dimensional maps of these ligands revealed that the ligand **12a** formed five interactions with the residues, Arg204, Asp340, Arg344, His296 and Asp206. Hydrogen bonds were observed between the residues Arg204, Arg344 and Asp206 with the oxygen atoms of groups CO, SO_2_ and OH of the ligand **12a**, respectively (Figure 3). However, this ligand showed a dipole–dipole interaction via its Cl atom with Asp340 residue, while an arene-H interaction was also found with the residue His296. In a recent report, flavonoid-based isoxazoles also showed interactions with the same type of residues during in silico screening against α-amylase enzyme [65]. The ligand **12d** exhibited two interactions with the residue Asp340 using its NH and nitrophenyl group. The compound **12g** exhibited different types of interactions with TrpA83, TrpB83, Gly167, His80 and Asp297. Gly167 showed an arene-H interaction, while Asp297 interacted with the OH group of the ligand **12g**. The rest of the residues (TrpA83, TrpB83 and His80) were found to form hydrogen bonds with CO groups of the compound **12g**. The ligand **12i**, having the lowest docking score, interacted with two selected residues, ArgA204 and ArgB204, using oxygen atoms of SO_2_ and CO groups, respectively. Recently, Duhan and colleagues also observed similar binding modes during the in silico inhibition of α-amylase by a variety of thiazole-pyrazole hybrids [67]. The acarbose was also re-docked into the receptor pocket of the enzyme to validate the experimental results.

Molecular docking results showed that the benzothiazine nucleus and the acetamide group were found important regarding the binding interactions with the receptor enzymes (α-glucosidase and α-amylase). Moreover, all the polar as well as non-polar groups in the structures of the ligands showed significant interactions with the selected residues. Prominently, the groups like OH, CH_2_, Cl, Br, NH, NO_2_, CO, SO_2,_ and aryl participated effectively in the inhibition of α-glucosidase and α-amylase enzymes, as revealed by the 2D maps of the screened derivatives (Figure 2 and Figure 3). Compounds having low binding energies, small RMSD values, and important bindings with the selected residues were of interest.

#### 2.3.2. Molecular Dynamics (MD) Studies for Complex Stability Analysis

**Figure 4:**
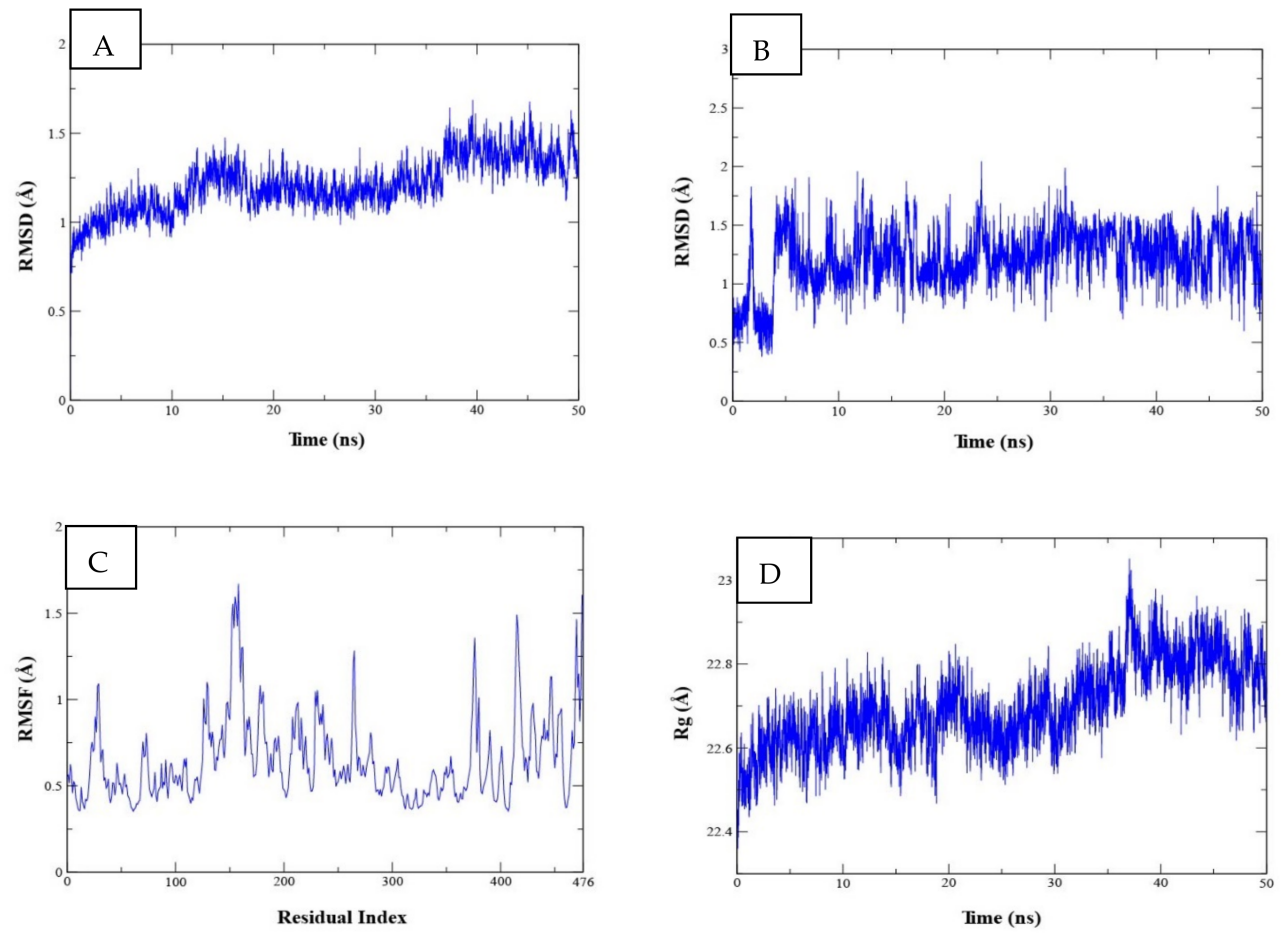
The MD simulation analysis of α-amylase during 50 ns. (**A**) shows the RMSD of protein backbone atoms. (**B**) shows the RMSD of ligand atoms. (**C**) shows the RMSF of each residue of protein. (**D**) shows the Rg of the protein backbone.

Molecular dynamics simulation studies were performed to investigate the binding stability of the most active compound, **12i**, with α-amylase protein. The RMSD value of the backbone of α-amylase was calculated to observe the complex stability during the simulation. In Figure 4A, backbone RMSD showed that protein was equilibrated at ~1 Å. The RMSD remained in the region of 1 Å until 10 ns and then reached ~1.35 Å; then, no change in behavior was observed till 36 ns. After that, a slight deviation of ~0.70 Å from ~38 to 50 ns took place. The overall behavior suggests that the compound was tightly bound with the protein in complex formation.

The ligand RMSD during simulation reached a maximum value of ~1.90 Å during the phase of 2 to 3 ns and then dropped to ~0.5 Å, as shown in Figure 4B. The average RMSD value of the ligand from the first binding pose remained ~1.25 Å during 50 ns simulations, which shows that compound **12i** made a stable complex with the protein.

To examine the fluctuations of amino acid residues of the complex, root mean square fluctuation (RMSF) was computed. The amino acid residues with low RMSF value are relatively more rigid than the residues with higher RMSF values. Figure 4C shows some minor and a major fluctuation at different amino acid residues. The minor fluctuations are at residual indexes of ~36, 75, 210, 270, 380 and 410. The major fluctuation was observed at the ~165 position. High RMSF values were found for those amino acids distant from the active site and the loops. The overall plot shows that very restricted fluctuations occurred at the binding site in the presence of the compound, showing that the binding pose had no clashes with the side chain of the α-amylase protein.

The radius of gyration (Rg) shows the compactness of the protein. The Rg value continues to rise or fall if unfolding events occur during the simulation. Figure 4D shows that the Rg plot of the α-amylase protein remains compact from 3 to ~12 ns, and then it decreases slightly. The Rg value during the entire 50 ns simulation remained the same, with less than 1 Å change from the original complex structure; this reveals that the compound is tightly bound in the active site with the right binding pose.

#### 2.3.3. Binding Free Energy Calculations

To examine the binding affinity of **12i** with α-amylase enzymes, an MM/GBSA model was used on last 50 frames of MD trajectory of protein-ligand complex. The total binding energy in terms of solvation energy, gas phase energy and entropic contributions was calculated (Table 2). The van der Waals and electrostatic energies were −50.65 ± 0.71 and −28.056 ± 1.56, respectively. The surface energy was −6.14, while the solvation and gas phase energies were 47.2406 ± 1.15 and −78.70 ± 2.11, respectively. The total binding free energy of the complex was −31.47 ± 1.07, which indicates that the ligand made a stable complex with the amylase enzyme.

### 2.4. Biological Activity

All the 1,2-benzothiazine derivatives were screened for their in vitro inhibitory potential against the α-glucosidase and α-amylase enzymes according to the reported protocols. PNPG (p-nitrophenyl-α-D-glucopyranoside) and starch were used as substrates, while acarbose was employed as a reference drug. Outcomes were found to be in correspondence with the in silico results. Initially, percentage inhibitions were calculated, and the derivatives with good percentage inhibitions (50 or above) were further evaluated to find the IC_50_ values via the micro dilution method (Table 1).

#### 2.4.1. α-Glucosidase Inhibition

**Figure 5:**
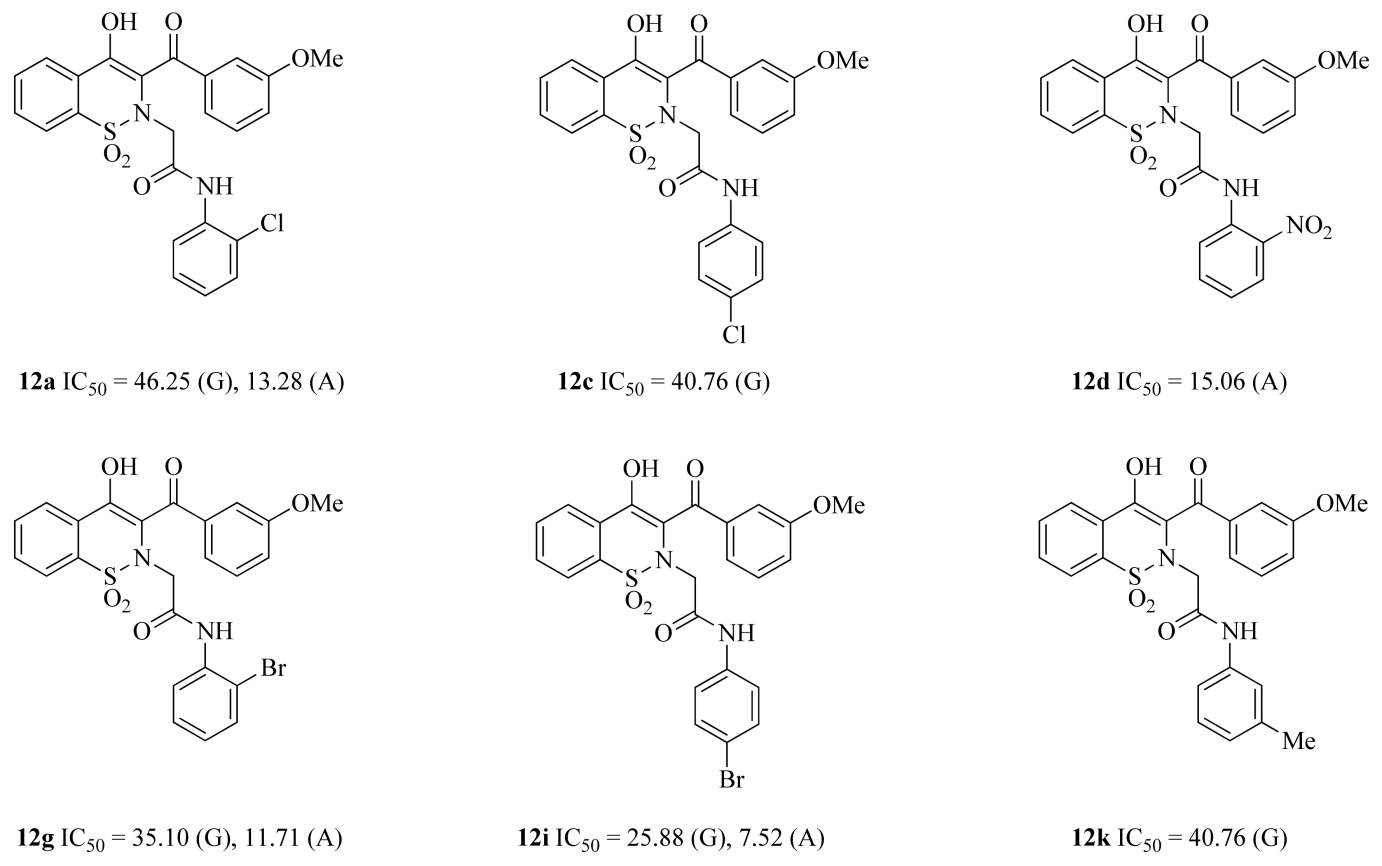
Structures of most potent compounds. IC_50_ values are in µM. (G) and (A) stands for α-Glucosidase and α-Amylase enzymes, respectively. Acarbose IC_50_ (µM) values = 58.8 (G), 17.0 (A).

Among the evaluated compounds, the derivatives **12a**, **12c**, **12g**, **12i** and **12k** exhibited prominent inhibition of the yeast α-glucosidase enzyme [68,69], having IC_50_ values of 46.25, 40.76, 35.10, 25.88 and 30.45 μM, respectively (Table 1, Figure 5), which were less than the reference drug, acarbose (IC_50_ = 58.8 μM [42,43]). Similarly, these derivatives were proven more effective α-glucosidase inhibitors in comparison to the reported benzimidazoles [70], dihydropyrano[2,3-c]pyrazoles [71] and 2-thioxobenzo[g]quinazolines [72] but less potent than the sulfonamide chalcones [44]. Similarly, compounds **12i** and **12k**, the most potent derivatives of the series, having IC_50_ values of 25.88 and 30.45 μM, respectively, were also found better α-glucosidase inhibitors compared to the recently reported *N*-arylacetamides [73]. These derivatives were found about 2.7- and 1.7-fold more potent enzyme inhibitors compared to the acarbose. As described previously during in silico screening, the compounds **12a**, **12c**, **12g**, **12i** and **12k** were found to be better α-glucosidase enzyme inhibitors. Hence, these derivatives were regarded as good anti-diabetic agents. Furthermore, the compounds **12f** and **12h**, with IC_50_ values of 97.03 and 86.21 μM, respectively, were observed as moderate α-glucosidase inhibitors, while the compounds **11**, **12b**, **12d**, **12e**, **12j**, **12l** and **12m** failed to show any remarkable inhibition of α-glucosidase enzyme and were categorized as ineffective compounds.

Regarding the structure–activity relationship, the presence of electron-withdrawing (Cl and Br) and electron-donating (like Me) groups resulted in better α-glucosidase inhibitions compared to others, as indicated by the low IC_50_ values of compound **12a** (46.25 μM), **12c** (40.76 μM), **12g** (35.10 μM), **12i** (25.88 μM) and **12k** (30.45 μM). Furthermore, it was noticed that the position of substituents (Cl, Br, Me) also had a significant effect on the enzyme inhibitory potentials of the derivatives. For example, the presence of halo groups (Cl, Br) at ortho and para positions of the *N*-phenyl moiety was found more effective compared to the meta position. This fact is justified by the low IC_50_ values of compounds **12a**, **12c**, **12g** and **12i** (Table 1). Similarly, the methyl substituent at the meta position provided a better enzyme inhibitory profile compared to other positions, which is indicated by the low IC_50_ value of derivative **12k**. In addition, the derivatives **12c** (40.76 μM) and **12i** (25.88 μM), having halogen (Cl, Br) substituents at the para position, showed more effective enzyme inhibitions than their isomeric derivatives **12a** (46.25 μM) and **12g** (35.10 μM), possessing halogen (Cl, Br) substituents at the ortho position. The presence of strongly activating and de-activating groups like nitro and methoxy, as in compounds **12d**, **12e**, **12f**, **12l** and **12m**, failed to show any promising inhibitory potential against the targeted enzyme.

#### 2.4.2. α-Amylase Inhibition

The 1,2-benzothiazine derivatives **11** and **12a–m** were subjected to in vitro analysis to evaluate their inhibitory potential against Aspergillus oryzae α-amylase enzyme [67,74]. The screened derivatives exhibited different extents of enzyme inhibition, with IC_50_ values ranging from 7.52 to 147.32 μM (Table 1). However, the derivatives **12a**, **12d**, **12g** and **12i** were the most active α-amylase inhibitors, with IC_50_ values of 13.28, 15.06, 11.71 and 7.52 μM (Table 1, Figure 5), respectively, which were more effective than the reference, acarbose (IC_50_ = 17.0 μM [75]). The derivatives **12e** and **12f** also exhibited good anti-diabetic potential, having IC_50_ values of 37.5 and 28.9 μM, respectively, while the remaining derivatives did not show any remarkable inhibition of α-amylase. 

The structure–activity relationship explained well the effect of the nature of substituents present on the *N*-phenyl ring regarding the inhibitory profiles of synthesized derivatives. It is noted that the derivatives having electron-withdrawing groups were proven better α-amylase inhibitors than the derivatives having electron-donating groups. This is justified by the low IC_50_ values of compounds **12a**, **12d**, **12g** and **12i**. It is further noticed that the electron-donating groups at ortho and para positions were proven fruitful regarding the inhibition of α-amylase, which is indicated by the low IC_50_ values of compounds **12a**, **12d**, **12f**, **12g** and **12i**. Furthermore, the derivative **12i** was witnessed as the most potent inhibitor of α-glucosidase enzymes as described above; herein, this derivative also proved the most effective inhibitor of α-amylase enzyme, having the lowest IC_50_ value (7.52 μM) of all other derivatives and about 2.5-fold smaller than the standard drug, acarbose. This derivative **12i** was also found to be a better α-amylase inhibitor as compared to the previously reported nicotinic acid derivatives [75], aryl imines [76], phosphoramidates [77] and piperazine sulfonamide analogs [45]. Moreover, the derivatives **12e** and **12f** also demonstrated good inhibitory potential against α-amylase, having IC_50_ values of 37.5 and 28.97 μM, respectively. The above discussion shows that the presence of chloro, bromo, nitro and methyl substituents in the *N*-phenyl group proved effective regarding the anti-diabetic potential of these scaffolds.

#### 2.4.3. Enzyme Inhibition Kinetics

**Figure 6:**
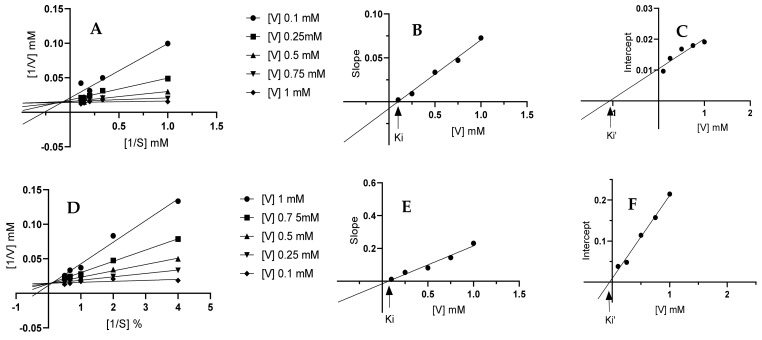
Kinetic analysis of α-glucosidase (**A**–**C**) and α-amylase (**D**–**F**) inhibition against various concentrations of **12i**. Enzyme reaction velocity (V, 1/V) is given in mM/min.

The pattern of the Lineweaver–Burk plot can visualize the type of inhibition, as shown in Figure 6A−F. The inverse of velocity (1/V) was taken on the *x*-axis and the inverse of substrate concentration (1/S) on the *y*-axis in the Lineweaver–Burk plot. This graph shows a straight line intersecting within first quadrant, illustrating the enzyme’s competitive inhibition. Secondary plots were drawn to determine the value of the inhibition constant Ki and the dissociation constant Ki’. Smaller Ki implies higher enzyme inhibition, implying the possibility of an allosteric binding site in the enzyme, to which synthetic drugs could bind. Compound **12i** prevents the enzyme competitively with a low value of Ki (0.0704) and Ki’ (−0.0564), which indicates the strong binding affinity of the inhibitor with enzyme α-amylase, while **12i** also inhibits the amylase, but in a non-competitive manner with Ki (0.1005) and Ki’ (−1.067) (Figure 6, Table 3). The computed results suggest a non-competitive-type inhibitory effect of alpha-glucosidase, as the Ki and Ki’ values in this plot differed from the reference drug (acarbose). 

Acarbose, a commercialized α-glucosidase inhibitor, is a competitive inhibitor of the enzyme that is used at greater concentrations to lower postprandial glycemic control. Compound **12i** acts as a non-competitive α-glucosidase inhibitor that interacts with the enzyme-substrate complex, reducing the Km and maximizing enzyme activity (Vmax). This type of inhibition shows that **12i** binds with free enzyme molecules and/or enzyme-substrate complexes. However, **12i** competitively inhibited the α-amylase enzyme, showing that **12i** inhibited the substrate from approaching the enzyme active site. 

## 3. Materials and Methods

### 3.1. General

An NMR-Spectrophotometer (Hazimin, 600 MHz, DMSO-*d_6_*) was used to record the ^1^H NMR and ^13^C NMR spectra (Supplementary Data). δ (Chemical shift) values are given in parts per million (ppm) in reference to tetramethylsilane (TMS). *J*-values (coupling constants) are written in Hertz (Hz). HRMS (ESI) spectra were recorded using an Agilent LC/TOF Mass 6210 Mass Spectrometer (Supplementary Data). Reaction progress was determined using silica-coated TLC plates (Merk, 0.2 mm 60 HF254), and a UV lamp (254 nm) was used to visualize the spots. 

*(4-Hydroxy-1,1-dioxido-2H-benzo[*e][1,2]*thiazin-3-yl)(3-methoxyphenyl)methanone* (**12**) [46,47]: Light yellow solid; yield: (242 mg, 73%); m.p. 153 °C (Literature m.p. 152–154 °C) [46].

### 3.2. General Procedure for the Synthesis of 2-(3-(3-methoxybenzoyl)-4-hydroxy-1,1-dioxido-2H-benzo[e][1,2] thiazin-2-yl)-N-arylacetamides (***12a–m***)

A mixture of 3-(3-Methoxybenzoyl)-1,2-benzothazine (**11**) (331 mg, 1.0 mmol) and 2-bromo-*N*-arylacetamides (1.0 mmol) under basic conditions (K_2_CO_3_, 207 mg, 1.5 mmols) in DMF (10 mL) was heated at 80–100 °C for 2–2.5 h. Cooling and dilution of the reaction mixture with cold distilled water followed by addition of cold dil. HCl (20%) yielded the precipitates of targeted 2-(3-(3-methoxybenzoyl)-4-hydroxy-1,1-dioxido-2*H*-benzo[e][1,2] thiazin-2-yl)-*N*-arylacetamides (**12a–m**) (Table 1). Precipitates were filtered, washed with an excess amount of distilled water, and then dried. Re-crystallization in methanol provided the pure compounds.

*N-(2-Chlorophenyl)-2-(4-hydroxy-3-(3-methoxybenzoyl)-1,1-dioxido-2H-benzo[*e][1,2]*thiazin-2-yl)acetamide* (**12a**) [78]: Yellow solid; yield: (409 mg, 82%); m.p. 186–187 °C. ^1^H NMR (DMSO-*d_6_*, 600 MHz) δ: 3.83 (s, 3H, OC*H_3_*), 4.05 (s, 2H, C*H_2_*), 7.24–7.26 (m, 1H, Ar*H*), 7.49 (t, *J* = 7.8 Hz, 1H, Ar*H*), 7.52–7.57 (m, 2H, Ar*H*), 7.57–7.58 (m, 1H, Ar*H*), 7.64 (d, *J* = 7.6 Hz, 1H, Ar*H*), 7.83–7.84 (m, 1H, Ar*H*), 7.90–7.96 (m, 3H, Ar*H*), 8.16–8.17 (m, 1H, Ar*H*), 8.20–8.22 (m, 1H, Ar*H*), 10.37 (s, 1H, N*H*). ^13^C NMR δ: 53.9, 55.2, 112.9, 113.8, 116.2, 117.9, 118.7, 120.9, 122.0, 124.8, 127.1, 128.3, 129.8, 130.1, 132.8, 133.9, 136.3, 138.3, 139.1, 147.7, 159.0, 165.5, 167.7, 189.1. MS (ESI^+^): *m/z* calcd. for C_24_H_19_ClN_2_O_6_NaS [M + Na]^+^ 521.0597; found 521.0892.

*N-(3-Chlorophenyl)-2-(4-hydroxy-3-(3-methoxybenzoyl)-1,1-dioxido-2H-benzo*[e][1,2]*thiazin-2-yl)acetamide* (**12b**): Light orange solid; yield: (424 mg, 85%); m.p. 142–143 °C. ^1^H NMR (DMSO-*d_6_*, 600 MHz) δ: 3.81 (s, 3H, OC*H_3_*), 4.07 (s, 2H, C*H_2_*), 7.23 (dd, *J =* 8.2, 2.0 Hz, 1H, Ar*H*), 7.47 (t, *J =* 8.0 Hz, 1H, Ar*H*), 7.54–7.58 (m, 2H, Ar*H*), 7.60 (t, *J =* 7.5, 2H, Ar*H*), 7.89–7.97 (m, 3H, Ar*H*), 8.14 (d, *J =* 7.8 Hz, 2H, Ar*H*), 8.21–8.24 (m, 1H, Ar*H*), 10.43 (s, 1H, N*H*). ^13^C NMR δ: 53.7, 55.3, 113.0, 116.4, 118.1, 118.8, 120.7, 121.9, 124.6, 127.2, 128.4 (2C), 129.7, 131.6 (2C), 134.0 (2C), 136.2, 138.4, 139.3, 146.2, 165.1, 167.1, 188.9. HRMS (ESI): *m/z* calcd. for C_24_H_19_ClN_2_O_6_NaS [M + Na]^+^ 521.0597; found 521.0894.

*N-(4-Chlorophenyl)-2-(4-hydroxy-3-(3-methoxybenzoyl)-1,1-dioxido-2H-benzo*[e][1,2]*thiazin-2-yl)acetamide* (**12c**): Yellow solid; yield: (394 mg, 79%); m.p. 146–147 °C. ^1^H NMR (DMSO-*d_6_*, 600 MHz) δ: 3.84 (s, 3H, C*H_3_*), 4.09 (s, 2H, C*H_2_*), 7.25–7.27 (m, 1H, Ar*H*), 7.45–7.47 (m, 2H, Ar*H*), 7.53–7.56 (m, 2H, Ar*H*), 7.63 (d, *J* = 7.6 Hz, 1H, Ar*H*), 7.89–7.96 (m, 3H, Ar*H*), 8.09–8.12 (m, 2H, Ar*H*), 8.19–8.21 (m, 1H, Ar*H*), 10.48 (s, 1H, N*H*). ^13^C NMR δ: 54.0, 55.3, 113.8, 116.1, 118.6 (2C), 118.7, 120.9, 121.8, 124.9 (2C), 127.1, 128.2, 129.8, 132.8, 133.9, 136.4, 138.5, 142.2, 144.2, 159.0, 165.9, 167.4, 189.3. HRMS (ESI): *m/z* calcd. for C_24_H_19_ClN_2_O_6_NaS [M(^37^Cl) + Na]^+^ 523.0597; found 523.0548.

*2-(4-Hydroxy-3-(3-methoxybenzoyl)-1,1-dioxido-2H-benzo*[e][1,2]*thiazin-2-yl)- N-(2-nitrophenyl)acetamide* (**12d**): Light orange solid; yield: (433 mg, 85%); m.p. 143–144 °C. ^1^H NMR (DMSO-*d_6_*, 600 MHz) δ: 3.84 (s, 3H, OC*H_3_*), 4.07 (s, 2H, C*H_2_*), 7.25–7.31 (m, 2H, Ar*H*), 7.40 (d, *J* = 8.2 Hz, 1H, Ar*H*), 7.54 (d, *J* = 8.0 Hz, 2H, Ar*H*), 7.56–7.59 (m, 1H, Ar*H*), 7.62 (d, *J* = 7.6 Hz, 1H, Ar*H*), 7.85–7.88 (m, 2H, Ar*H*), 7.89–7.90 (m, 2H, Ar*H*), 8.13–8.16 (m, 1H, Ar*H*), 10.17 (s, 1H, N*H*), 15.01 (s, 1H, O*H*enolic). ^13^C NMR δ: 53.6, 55.2, 113.6, 116.1, 118.6, 120.7, 121.9, 124.8, 125.1, 125.5, 127.1, 128.3, 129.9, 130.2, 132.7, 133.8, 133.9, 136.3, 138.2, 141.7, 159.0, 165.5, 168.3, 188.7. HRMS (ESI): *m/z* calcd. for C_24_H_19_N_3_O_8_NaS [M + Na]^+^ 532.0797; found 532.0331.

*2-(4-Hydroxy-3-(3-methoxybenzoyl)-1,1-dioxido-2H-benzo*[e][1,2]*thiazin-2-yl)- N-(3-nitrophenyl)acetamide* (**12e**): Light orange solid; yield: (423 mg, 83%); m.p. 189–190 °C. ^1^H NMR (DMSO-*d_6_*, 600 MHz) δ: 3.82 (s, 3H, OC*H_3_*), 4.08 (s, 2H, C*H_2_*), 7.02–7.13 (m, 2H, Ar*H*), 7.19 (dd, *J =* 8.3, 2.1 Hz, 2H, Ar*H*), 7.34–7.40 (m, 1H, Ar*H*), 7.75 (t, *J* = 8.2 Hz, 1H, Ar*H*), 7.92–8.00 (m, 2H, Ar*H*), 8.04 (d, *J* = 7.6 Hz, 2H, Ar*H*), 8.24–8.29 (m, 2H, Ar*H*), 10.12 (s, 1H, N*H*). ^13^C NMR δ: 53.7, 55.3, 114.1, 117.8, 118.7, 119.4, 121.0, 121.7, 126.3, 126.8, 127.4 (2C), 127.6, 129.1, 132.6, 133.7, 134.9, 136.2, 138.0, 141.9, 159.3, 164.9, 167.6, 188.1. HRMS (ESI): *m/z* calcd. for C_24_H_19_N_3_O_8_NaS [M + Na]^+^ 532.0797; found 532.0808.

*2-(4-Hydroxy-3-(3-methoxybenzoyl)-1,1-dioxido-2H-benzo[e]*[1,2]*thiazin-2-yl)- N-(4-nitrophenyl)acetamide* (**12f**): Orange solid; yield: (407 mg, 80%); m.p. 176–177 °C. ^1^H NMR (DMSO-*d_6_*, 600 MHz) δ: 3.85 (s, 3H, OC*H_3_*), 4.06 (s, 2H, C*H_2_*), 7.21–7.28 (m, 3H, Ar*H*), 7.70 (t, *J =* 7.3 Hz,1H, Ar*H*), 7.73 (t, *J =* 7.6 Hz, 2H, Ar*H*), 7.85–7.90 (m, 1H, Ar*H*), 7.92–7.94 (m, 2H, Ar*H*), 8.05 (d, *J =* 7.7 Hz, 2H, Ar*H*), 8.19–8.21 (m, 1H, Ar*H*), 10.11 (s, 1H, N*H*). ^13^C NMR δ: 53.8, 55.7, 113.9, 116.2, 121.2, 121.8, 127.0, 127.9, 128.4, 128.5, 128.6, 128.7 (2C), 132.7, 132.9, 133.8, 136.2, 137.0, 138.5, 142.0, 159.1, 165.9, 167.9, 188.6. HRMS (ESI): *m/z* calcd. for C_24_H_19_N_3_O_8_NaS [M + Na]^+^ 532.0797; found 532.0612.

*N-(2-Bromophenyl)-2-(4-hydroxy-3-(3-methoxybenzoyl)-1,1-dioxido-2H-benzo*[e][1,2]*thiazin-2-yl)acetamide* (**12g**): Yellow solid; yield: (467 mg, 86%); m.p. 169–171 °C. ^1^H NMR (DMSO-*d_6_*, 600 MHz) δ: 3.73 (s, 3H, OC*H_3_*), 4.11 (s, 2H, C*H_2_*), 6.73–6.76 (m, 1H, Ar*H*), 6.93 (dd, *J* = 7.8, 1.4 Hz, 1H, Ar*H*), 6.96–6.99 (m, 1H, Ar*H*), 7.51 (d, *J* = 7.8 Hz, Ar*H*), 7.84 (d, *J* = 7.8 Hz, Ar*H*), 7.86–7.91 (m, 4H, Ar*H*), 7.94 (d, *J* = 7.8 Hz, 2H, Ar*H*), 8.16–8.18 (m, 1H, Ar*H*), 9.17 (s, 1H, N*H*), 14.92 (s, 1H, O*H*enolic). ^13^C NMR δ: 54.01, 55.57, 111.0, 116.5, 120.1, 121.2, 122.0, 124.4, 126.4, 127.0, 127.1, 128.5, 130.7, 131.8, 132.7, 133.9, 134.3, 138.4, 149.1, 165.0, 168.0, 187.9. HRMS (ESI): *m/z* calcd. for C_24_H_19_BrN_2_O_6_NaS [M(^81^Br) + Na]^+^ 566.9997; found 566.9809.

*N-(3-Bromophenyl)-2-(4-hydroxy-3-(3-methoxybenzoyl)-1,1-dioxido-2H-benzo*[e][1,2]*thiazin-2-yl)acetamide* (**12h**): Yellow powder; yield: (434 mg, 80%); m.p. 132–133 °C. ^1^H NMR: (DMSO-*d_6_*, 600 MHz)δ: 3.87 (s, 3H, OC*H_3_*), 4.05 (s, 2H, C*H_2_*), 7.17 (d, *J* = 7.9 Hz, 2H, Ar*H*), 7.21–7.34 (m, 2H, Ar*H*), 7.56 (d, *J* = 7.9 Hz, 3H, Ar*H*), 7.67–7.75 (m, 2H, Ar*H*), 7.96 (t, *J* = 7.9 Hz, 1H, Ar*H*), 8.01–8.06 (m, 1H, Ar*H*), 8.13–8.17 (m, 1H, Ar*H*), 9.59 (s, 1H, N*H*). ^13^C NMR δ: 53.8, 55.4, 113.3, 116.2, 117.9, 119.8, 121.63, 124.3, 125.5, 126.0, 127.3, 128.3, 129.7, 130.3, 132.7, 133.4, 136.6 (2C), 138.5, 139.9, 159.2, 164.4, 167.9, 188.4. HRMS (ESI): *m/z* calcd. for C_24_H_19_BrN_2_O_6_NaS [M + Na]^+^ 564.9997; found 564.9973.

*N-(4-Bromophenyl)-2-(4-hydroxy-3-(3-methoxybenzoyl)-1,1-dioxido-2H-benzo*[e][1,2]*thiazin-2-yl)acetamide* (**12i**): Light yellow solid; yield: (429 mg, 79%); m.p. 140 °C. ^1^H NMR (DMSO-*d_6_*, 600 MHz)δ: 3.84 (S, 3H, OC*H_3_*), 4.01 (s, 2H, C*H_2_*), 7.14–7.17 (m, 3H, Ar*H*),7.26 (dd, *J =* 7.8, 2.7 Hz,1H, Ar*H*), 7.48–7.49 (m, 1H, Ar*H*), 7.55 (t, *J =* 7.8 Hz, 2H, Ar*H*), 7.63 (d, *J =* 7.6 Hz, 1H, Ar*H*), 7.88–7.95 (m, 3H, Ar*H*), 8.18–8.21 (m, 1H, Ar*H*), 10.04 (s, 1H, N*H*). ^13^C NMR δ: 53.8, 55.3, 113.8, 116.2, 117.7, 118.7, 120.9, 121.1, 121.3, 121.9, 126.0, 127.1, 128.3, 129.8, 130.6, 132.8, 133.9, 136.3, 138.4, 139.6, 159.0, 165.1, 167.7, 189.0. HRMS (ESI): *m/z* calcd. for C_24_H_19_BrN_2_O_6_NaS [M + Na]^+^ 564.9997; found 564.9971.

*2-(4-Hydroxy-3-(3-methoxybenzoyl)-1,1-dioxido-2H-benzo*[e][1,2]*thiazin-2-yl)-N-(o-tolyl)acetamide* (**12j**): Yellow powder; yield: (373 mg, 78%); m.p. 166 °C. ^1^H NMR (DMSO-*d_6_*, 600 MHz) δ: 2.18 (s, 3H, C*H_3_*), 3.89 (s, 3H, OC*H_3_*), 4.01 (s, 2H, C*H_2_*), 6.80 (t, *J =* 7.8 Hz, 2H, Ar*H*), 7.03 (dd, *J =* 7.3, 2.3 Hz, 2H Ar*H*), 7.24–7.27 (m, *J =* 7.8 Hz, 2H, Ar*H*), 7.58 (dd, *J =* 7.8, 2.7 Hz, 1H, Ar*H*), 7.65 (d, *J =* 7.8 Hz, 1H, Ar*H*), 7.85–7.90 (m, 1H, Ar*H*), 8.17 (d, 1H, Ar*H*), 9.74 (s, 1H, N*H*). ^13^C NMR δ: 21.2, 54.0, 55.1, 113.6, 116.3, 116.4, 118.6, 119.0, 120.7, 121.5, 126.4, 127.4 (2C), 128.7 (2C), 130.0, 132.6, 133.9, 136.7, 138.0, 138.2, 139.1, 165.2, 167.2, 188.9. HRMS (ESI): *m/z* calcd. for C_25_H_22_N_2_O_6_HS [M + H]^+^ 479.1097; found 479.1151.

*2-(4-Hydroxy-3-(3-methoxybenzoyl)-1,1-dioxido-2H-benzo*[e][1,2]*thiazin-2-yl)-N-(m-tolyl)acetamide* (**12k**): Light yellow solid; yield: (378 mg, 79%); m.p. 150–151 °C. ^1^H NMR (DMSO-*d_6_*, 600 MHz) δ: 2.16 (s, 3H, C*H_3_*), 3.86 (s, 3H, OC*H_3_*), 4.01 (s, 2H, C*H_2_*), 6.79 (d, *J =* 7.2 Hz, 1H, Ar*H*), 7.02–7.07 (m, 2H, Ar*H*), 7.28 (dd, *J =* 7.8, 2.7 Hz, 1H, Ar*H*), 7.54–7.58 (m, *J =* 7.8 Hz, 2H, Ar*H*), 7.66 (d, *J =* 7.6 Hz, 1H, Ar*H*), 7.88–7.93 (m, 4H, Ar*H*), 8.19–8.20 (m, 1H, Ar*H*), 9.78 (s, 1H, N*H*). ^13^C NMR δ: 21.0, 53.7, 55.2, 113.8, 116.1, 116.2, 118.7, 119.4, 120.9, 121.8, 124.1, 127.1, 128.4 (2C), 129.8, 132.7, 133.8, 136.4, 137.8, 138.0, 138.5, 159.0, 164.6, 167.9, 188.8. HRMS (ESI): *m/z* calcd. for C_25_H_22_N_2_O_6_NaS [M + Na]^+^ 501.1097; found 501.1467.

*2-(4-Hydroxy-3-(3-methoxybenzoyl)-1,1-dioxido-2H-benzo*[e][1,2]*thiazin-2-yl)- N-(2-methoxyphenyl)acetamide* (**12l**): Yellow solid; yield: (420 mg, 85%); m.p. 190–191 °C. ^1^H NMR: (DMSO-*d_6_*, 600 MHz) δ: 3.72 (s, 3H, OC*H_3_*), 3.84 (s, 3H, OC*H_3_*), 4.10 (s, 2H, C*H_2_*), 6.74 (t, *J =* 8.2 Hz, 1H, Ar*H*), 9.93 (dd, *J =* 8.2, 1.5 Hz, 1H, Ar*H*), 6.98 (t, *J =* 7.8 Hz, 1H, Ar*H*), 7.27 (dd, *J =* 8.2, 2.7 Hz, 1H, Ar*H*), 7.50 (d, *J =* 7.8 Hz, 1H, Ar*H*), 7.54 (d, *J =* 7.8 Hz, 1H, Ar*H*), 7.57 (d, *J =* 9.6 Hz, 1H, Ar*H*), 7.64 (d, *J =* 7.2 Hz, 1H, Ar*H*), 7.86–7.91 (m, 3H, Ar*H*), 8.16–8.18 (m, 1H, Ar*H*), 9.16 (s, 1H, N*H*). ^13^C NMR δ: 53.8, 55.2, 55.5, 111.1, 113.7, 116.4, 118.7, 120.0, 120.9, 121.3, 121.8, 124.4, 126.4, 127.0, 128.5, 129.8, 132.6, 133.7, 136.3, 138.5, 149.2, 159.0, 165.1, 168.0, 188.6. HRMS (ESI): *m/z* calcd. for C_25_H_22_N_2_O_7_NaS [M + Na]^+^ 517.0997; found 517.1576.

*2-(4-Hydroxy-3-(3-methoxybenzoyl)-1,1-dioxido-2H-benzo*[e][1,2]*thiazin-2-yl)- N-(4-methoxyphenyl)acetamide* (**12m**): Yellow solid; yield: (410 mg, 83%); m.p. 201–202 °C. ^1^H NMR (DMSO-*d_6_*, 600 MHz) δ: 3.65 (s, 3H, OC*H_3_*), 3.84 (s, 3H, OC*H_3_*), 3.97 (s, 2H, C*H_2_*), 6.74–6.77 (m, 2H, Ar*H*), 7.12 (d, *J =* 8.4 Hz, 2H, Ar*H*), 7.27 (dd, *J =* 7.8, 2.7, 1H, Ar*H*), 7.55 (t, *J =* 7.8 Hz, 2H, Ar*H*), 7.64 (d, *J =* 7.6, 1H, Ar*H*), 7.87–7.93 (m, 3H, Ar*H*), 8.17–8.19 (m, 1H, Ar*H*), 9.73 (s, 1H, N*H*). ^13^C NMR δ: 53.7, 55.0, 55.2, 113.7 (2C), 113.8, 116.2, 118.7, 120.4, 120.9, 121.8, 127.0, 128.4, 129.9, 131.2, 132.6, 133.8, 136.3, 138.5, 155.2, 155.6, 159.0, 164.1, 168.1, 188.7. HRMS (ESI): *m/z* calcd. for C_25_H_22_N_2_O_7_NaS [M + Na]^+^ 517.0997; found 517.1573.

### 3.3. In Silico Analysis

#### 3.3.1. Molecular Docking

Molecular docking of the synthesized 1,2-benzothiazine derivatives along with reference acarbose against α-glucosidase and α-amylase enzymes was performed via MOE software, Ver.2014.0901 [79,80,81]. ChemDraw Ultra 12.02 was utilized to sketch the structures of all the compounds, and these structures were saved in MDL file (“.sdf”) to display in MOE. The two-dimensional structure of the standard drug, acarbose, was downloaded from NCBI Pubchem in ‘sdf’ format. The three-dimensional structures of α-glucosidase *(*N-terminal maltase glucoamylase (PDB ID: 2QMJ)) and *Aspergillus oryzae* α-amylase (PDB ID: 7TAA) were retrieved from the Protein Data Bank (http://www.rcsb.org/pdb, accessed on 20 June 2021). Interaction modes of the bound ligand (acarbose) with α-glucosidase residues (Asp203, Asp542, Asp327, His600, Arg526) and α-amylase residues (Trp83, Asp340, Arg344, Arg204, Glu230, Lys209) were observed. All the ligands, reference acarbose and the receptor protein were 3D-protonated and energy was minimized with the help of the MMFF94X force field. Docking of all the synthesized compounds and acarbose (ten conformations of each) into the targeted sites of the receptor enzymes was executed by setting the parameters—rescoring: London dG, placement: triangle matcher, retain: 10, and refinement: forcefield. RMSD values, binding energies and binding modes with the selected residues were considered to identify the leading conformations of the docked ligands. 2D-docking modes are given in Figure 2 and Figure 3 while 3D-docking modes are presented in Figures S1 and S2 (Supplementary Data).

#### 3.3.2. MD Simulation

To check the stability and refinement of the structure, the amylase complex was subjected to MD simulation. The simulation was performed in a solvation system using the NAMD tool. The files were prepared by AMBER20 tools. In file preparation, the missing hydrogens were added by the Leap program [82] to the protein. The amylase complex was solvated in a periodic cubic box of TIP3P water molecules [83] with a box size of 10 Å. The solvation box was then neutralized by replacing Na^+^ and Cl^-^ counter ions with water molecules using the Leap program. The force field parameters for the protein and ligand were the AMBER ff14SB force field [84] and general amber force field, respectively. The system was relaxed by an energy minimization step prior to MD simulation to avoid any stearic clashes. The energy was minimized using 10,000 steps. After minimization, the solvation equilibration of the system was done at 300 K. For maintaining system stability, three equilibration steps at different temperatures, i.e., 200 k, 250 k and 300 k, were performed. Ultimately, the system was employed to run MD simulation for 20 ns with constant temperature 300 K and a pressure of 1 atm. All the bonds containing hydrogen atoms were constrained using the SHAKE algorithm [85] at 2 fs time. The particle-mesh-Ewald (PME) method [86] was applied to calculate the electrostatic interactions with a cutoff of 10 Å to treat non-bonded interactions. The MD trajectory was stored at every 2 ps during the production run. The analysis of the MD trajectory was carried out using VMD, R package, and CPPTRAJ modules of AMBER20 tools. 

#### 3.3.3. Binding Free Energy Calculations

The binding free energy of the system was calculated by using the molecular mechanics-based scoring methods MM/GB(PB)SA. The calculations were based on a total of 1000 snapshots of the complex, taken at 2 ps intervals from the last 2 ns stable MD trajectory. The binding free energy was determined as the difference between the total free energy (ΔG_com_) of the ligand-receptor complex and the sum of free energy of individual receptors (ΔG_pro_) and ligands (ΔG_lig_) using the equation provided below:

ΔG_bind_ = ΔH − TΔS = ΔG_com_ − (ΔG_pro_ + ΔG_lig_)

### 3.4. Biological Activity

#### 3.4.1. α-Glucosidase Inhibition

α-Glucosidase inhibitions were determined following a modified spectroscopic method [87]. A solution of α-glucosidase (obtained from *Saccharomyces cerevisiae* (Sigma-Aldrich, Munich, Germany)) was prepared in phosphate buffer (pH 6.8, 100 mM). Wells of the 96-well microliter plates were filled with test sample solutions (12.5 μL) prepared in DMSO. After this, enzyme (0.5 U/mL, 40 μL) and then phosphate buffer (100 mM, 120 μL) were also mixed with the test sample solutions. Incubation at 37 °C for 5 min and then addition of 40 μL of the substrate, *p*-nitrophenyl-α-D-glucopyranoside (PNPG) (5 nM, Sigma-Aldrich, Munich, Germany), was performed. Mixtures were again incubated for 30 min, and absorbance was measured at 405 nm and 37 °C for the released 4-nitrophenol in each mixture. Acarbose (Sigma-Aldrich, Munich, Germany) and DMSO worked as standard and negative inhibitors, respectively. Each experiment was carried out thrice, and percentage inhibitions were determined with the help of following expression, followed by the determination of IC_50_ values.

% Inhibition = [(A_control_ − A_sample_)/A_control_] × 100

where A is absorbance.

#### 3.4.2. α-Amylase Inhibition

A previously reported protocol [88,89,90] was followed, with modifications, to assess the α-amylase inhibitory potential of all the synthesized compounds along with standard acarbose. A total of 40 µL of each test sample was pipetted out in 96-well microliter plates. This was mixed with 40 µL of *Aspergillus oryzae* α-amylase (Sigma-Aldrich, Munich, Germany) solution (prepared in Na_2_SO_4_ buffer (0.02 M, pH = 6.9)). Incubation of solutions was executed at 37 °C for 10 min. After incubation, 40 µL of starch solution prepared in 1% DMSO was added into each well as a substrate and placed at 25 °C for 10 min. After this, 100 µL of 3,5-dinitrosalicylic acid (DNSA) was added and incubated for 5 min in boiling water. After cooling and dilution of mixtures with distilled water (400 μL), the absorbance was recorded at 540 nm wavelength [91] and 37 °C temperature with the help of a microplate reader. Acarbose (Sigma-Aldrich, Munich, Germany) and DMSO worked as standard and negative inhibitors, respectively. Each experiment was carried out thrice, and percentage inhibitions were determined with the help of following expression, followed by the determination of IC_50_ values.

% Inhibition = [(A_control_ − A_sample_)/A_control_] × 100

where A is absorbance.

#### 3.4.3. Enzyme Inhibition Kinetics

The Lineweaver–Burk equation was used to determine the type of inhibition. α-Glucosidase and α-amylase activities were studied in the absence and presence of various sample concentrations (0.1 mM, 0.25 mM, 0.5 mM, 0.75 mM and 1.0 mM). For α-glucosidase inhibition kinetics, PNPG was used as a substrate, with different concentrations (1 mM, 3 mM, 5 mM, 7 mM and 9 mM). In α-amylase inhibition kinetics, various amounts of starch solution (0.25–2%) were tested. All experiments were carried out in triplicate, and the outcomes were written as mean ± S.D.

## 4. Conclusions

We presented 2-(3-(3-methoxybenzoyl)-4-hydroxy-1,1-dioxido-2*H*-benzo[*e*][1,2]thiazin-2-yl)-*N*-arylacetamides as potent inhibitors of α-glucosidase and α-amylase enzymes. The derivatives **12a**, **12c**, **12g**, **12i** and **12k** delivered the best in vitro α-glucosidase inhibitions, as indicated by their low IC_50_ values of 46.25, 40.76, 35.10, 25.88 and 30.45 μM, respectively, compared to the reference drug, acarbose (IC_50_ = 58.8 μM). Similarly, the derivatives **12a**, **12d**, **12g** and **12i** were proven the best inhibitors of α-amylase enzymes, having IC_50_ values of 13.28, 15.06, 11.71 and 7.52 μM, respectively, which were even lower than the acarbose (IC_50_ = 17.0 μM). Compound, *N*-(4-bromophenyl)-2-(4-hydroxy-3-(3-methoxybenzoyl)-1,1-dioxido-2*H*-benzo[e][1,2]thiazin-2-yl)acetamide (**12i**), the most potent derivative, showed competitive and non-competitive inhibitions of α-glucosidase and α-amylase enzyme, respectively. Molecular docking studies provided the structure–activity relationship and binding modes of the compounds with the targeted enzymes. The ligand–protein complex stability and binding energies were calculated using molecular dynamics throughout the 200 ns time. The RMSD values of ligands and RMSF values of active site residues indicated that the ligands formed a stable complex with the protein. In summary, the described templates are a good addition to the library of anti-diabetic agents.

## Figures and Tables

**Table 1:** Structural parameters and in silico (docking scores and binding residues) and in vitro (IC_50_ values) analysis of 1,2-benzothiaine-*N*-arylacetamides **12a–m** against α-glucosidase and α-amylase enzymes.

Compd.	Substituent	α-Glucosidase				α-Amylase			
Code	R	Docking Score (Kcal/mol)	RMSD Value (Å)	Binding Residues	IC_50_ (μM)	Docking Score (Kcal/mol)	RMSD Value (Å)	Binding Residues	IC_50_ (μM)
**11**	-	-		*-*	ND	-		-	147.32
**12a**	2-Cl	−12.50	1.28	Asp203, Thr205	46.25	−15.24	1.03	Arg204, Asp340	13.28
**12b**	3-Cl	-		*-*	ND	-		*-*	ND
**12c**	4-Cl	−12.80	1.29	Asp542	40.76	-		*-*	124.6
**12d**	2-NO_2_	-		-	102.33	−14.89	1.22	Asp340	15.06
**12e**	3-NO_2_	-		-	ND	-		-	37.5
**12f**	4-NO_2_	-		*-*	97.03	−14.60	-	-	28.97
**12g**	2-Br	−12.98	1.22	Asp542*,* Met444	35.10	−15.89	0.88	Trp83	11.71
**12h**	3-Br	-		*-*	86.21	-		*-*	109.6
**12i**	4-Br	−13.30	1.01	Asp327*,* Asp542	25.88	−16.13	0.75	Arg204	7.52
**12j**	2-Me	-		-	ND	-		*-*	ND
**12k**	3-Me	−13.22	1.12	Asp203	30.45	-		*-*	67.23
12l	2-OMe	-		*-*	110.23	-		*-*	ND
**12m**	4-OMe	-		*-*	ND	-		*-*	121.88
**Acarbose**	-	−16.18	2.12	His600, Asp542, Arg526, Asp327, Met444, Lys480	58.8	−17.69	2.01	Arg204, Glu230	17.0

ND = Not Determined; acarbose = reference drug.

**Table 2:** MM/GBSA calculated values for different parameters of protein–ligand complex.

Binding Energy Components	Amylase–Ligand	Std. Dev.	Std. Err. of Mean
ΔG_bind_ = (ΔG_complex_) − (ΔG_protein_ + ΔG_ligand_)			
MMGBSA			
VDWAALS	−50.6534	5.6170	0.7944
EEL	−28.0565	13.2835	1.8786
EGB	53.3866	10.3616	1.4654
ESURF	−6.1459	0.3115	0.0440
DELTA G gas	−78.7099	17.7063	2.5041
DELTA G solv	47.2406	10.1638	1.4374
DELTA TOTAL	−31.4693	8.6701	1.2261

**Table 3:** Kinetic parameters of α-glucosidase and α-amylase enzymes against **12i** and its various concentrations.

12i	Ki	Ki’
α-glucosidase	0.1005	−1.067
α-amylase	0.0704	−0.0564

## Data Availability

Data is contained within the article and Supplementary Material.

## Supplementary Materials

The following supporting information can be downloaded at: https://www.mdpi.com/article/10.3390/ph15010106/s1, ^1^H NMR, ^13^C NMR, HRMS (ESI) spectra and 3D docking modes (Figure S1, Figure S2) of potent compounds (**12a**, **12c**, **12d**, **12g**, **12i**, **12k**).
