# Supplementary material for: Identification of Cyclic Sulfonamides with an N-Arylacetamide Group as α-Glucosidase and α-Amylase Inhibitors: Biological Evaluation and Molecular Modeling
Source: Pharmaceuticals (Basel). 2022 Jan 17;15(1):106. doi: 10.3390/ph15010106 (PMC8777765; doi:10.3390/ph15010106)
Supplement: Supplementary file 1 [file pharmaceuticals-15-00106-s001.zip › pharmaceuticals-1471120-supplementary.pdf]

Identification of cyclic sulfonamides with an *N*-arylacetamide group as *α*-glucosidase and *α*-amylase inhibitors: Biological evaluation and molecular modeling

Furqan Ahmad Saddique [1], Matloob Ahmad [1,*], Usman Ali Ashfaq [2], Muhammad Muddassar [3], Sadia Sultan [4,5] and Magdi E. A. Zaki [6,*]

[1] Department of Chemistry, Government College University, Faisalabad 38000, Pakistan; furqanas123@gmail.com
[2] Department of Bioinformatics and Biotechnology, Government College University, Faisalabad 38000, Pakistan; ashfaqua@gcuf.edu.pk
[3] Department of Biosciences, COMSATS University Islamabad, Park Road, Islamabad 45500, Pakistan;
mmuddassar@comsats.edu.pk
[3] Faculty of Pharmacy, Universiti Teknologi MARA, Puncak Alam Campus, Bandar Puncak Alam 42300, Selangor Darul Ehsan, Malaysia; drsadia@uitm.edu.my
[4] Atta-ur-Rahman Institute for Natural Products Discovery (AuRIns), Universiti Teknologi MARA, Puncak Alam Campus, Bandar Puncak Alam 42300, Selangor Darul Ehsan, Malaysia; drsadia@uitm.edu.my
[5] Department of Chemistry, Faculty of Science, Imam Mohammad Ibn Saud Islamic University (IMSIU), Riyadh 11623, Saudi Arabia
* Correspondence: Matloob.Ahmad@gcuf.edu.pk (M.A.); mezaki@imamu.edu.sa (M.E.A.Z.)

(Supplementary Data File)

^{1}H NMR, ^{13}C NMR, HRMS (ESI) spectra and 3D docking modes (Figure S1, Figure S2) of potent compounds (**12a, 12c, 12d, 12g, 12i, 12k**). All the spectra were recorded using NMR Spectrophotometer (Hazimin) in deuterated DMSO and at 600 MHz frequency. HRMS (ESI) spectra were recorded using Agilent LC/TOF Mass 6210 Mass Spectrometer.

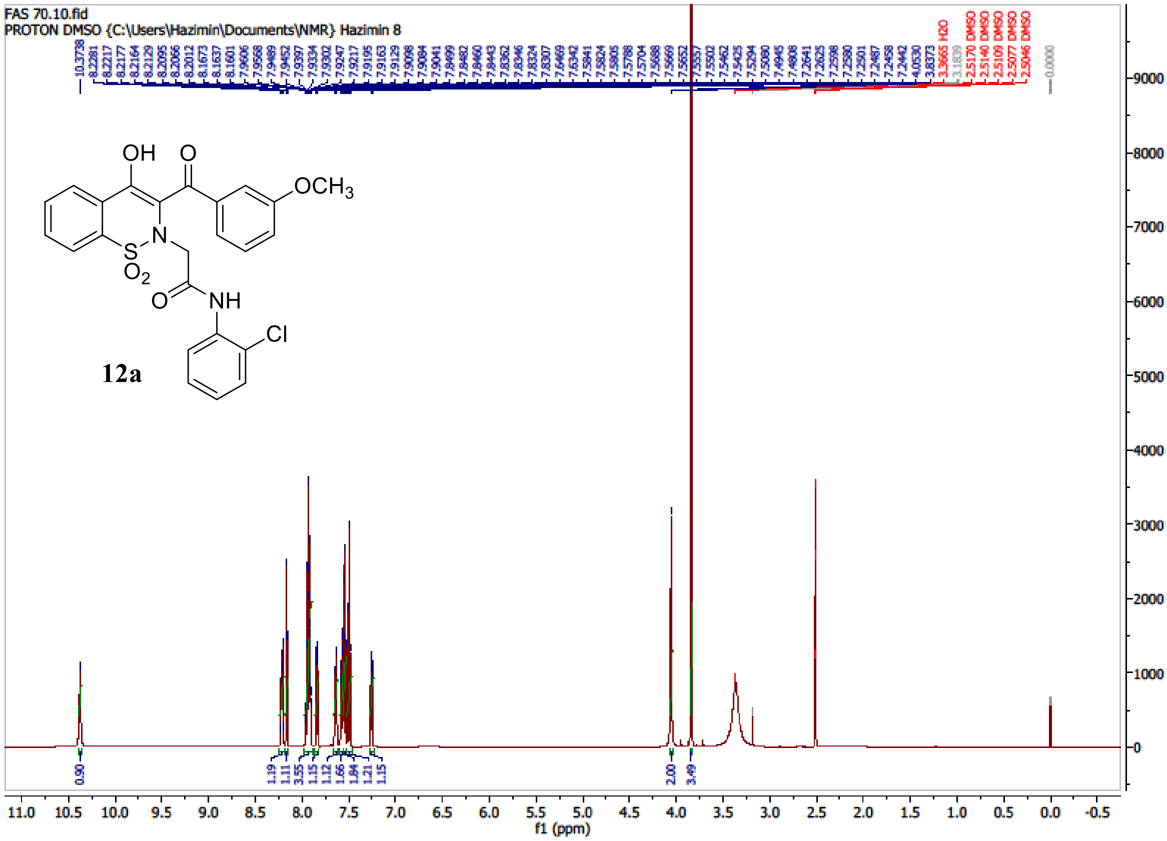

^{1}H NMR Spectrum of compound **12a**.

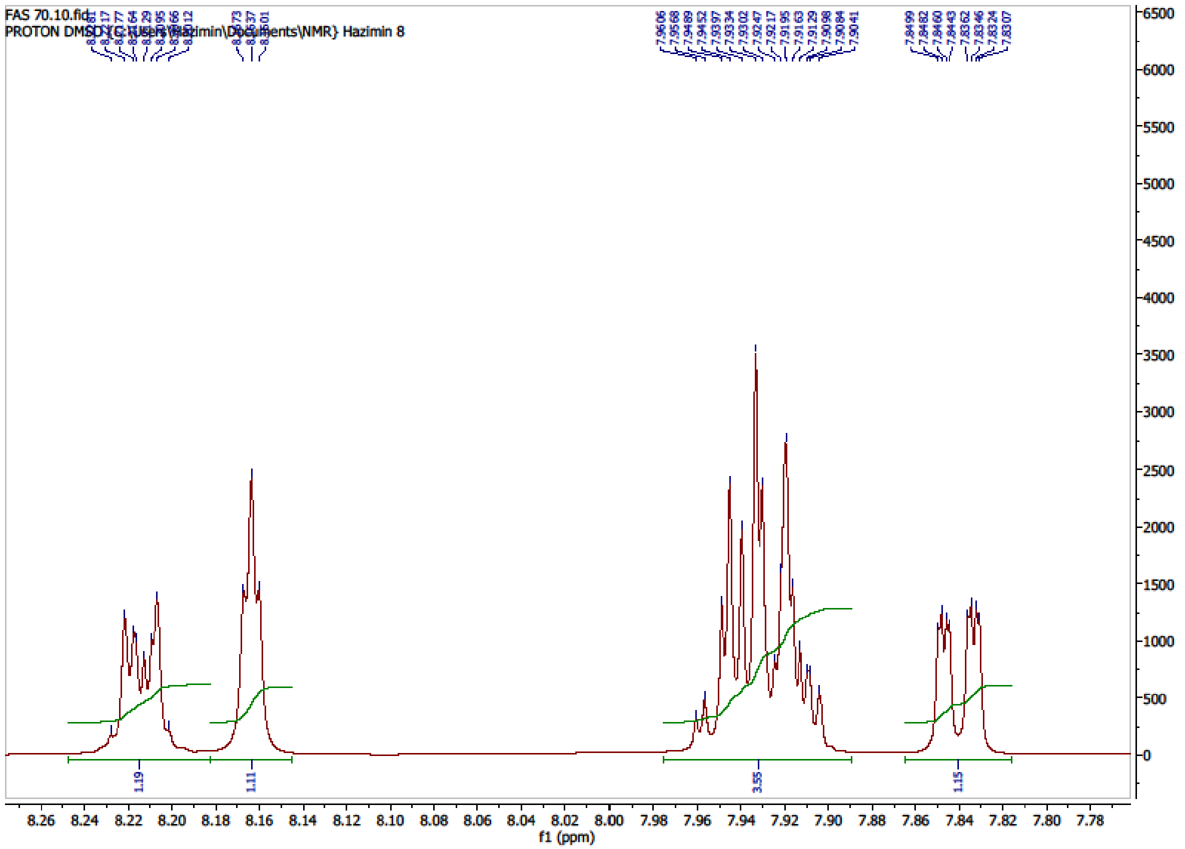

Expanded form of ¹H NMR Spectrum of compound **12a** (region 7.80-8.26 ppm).

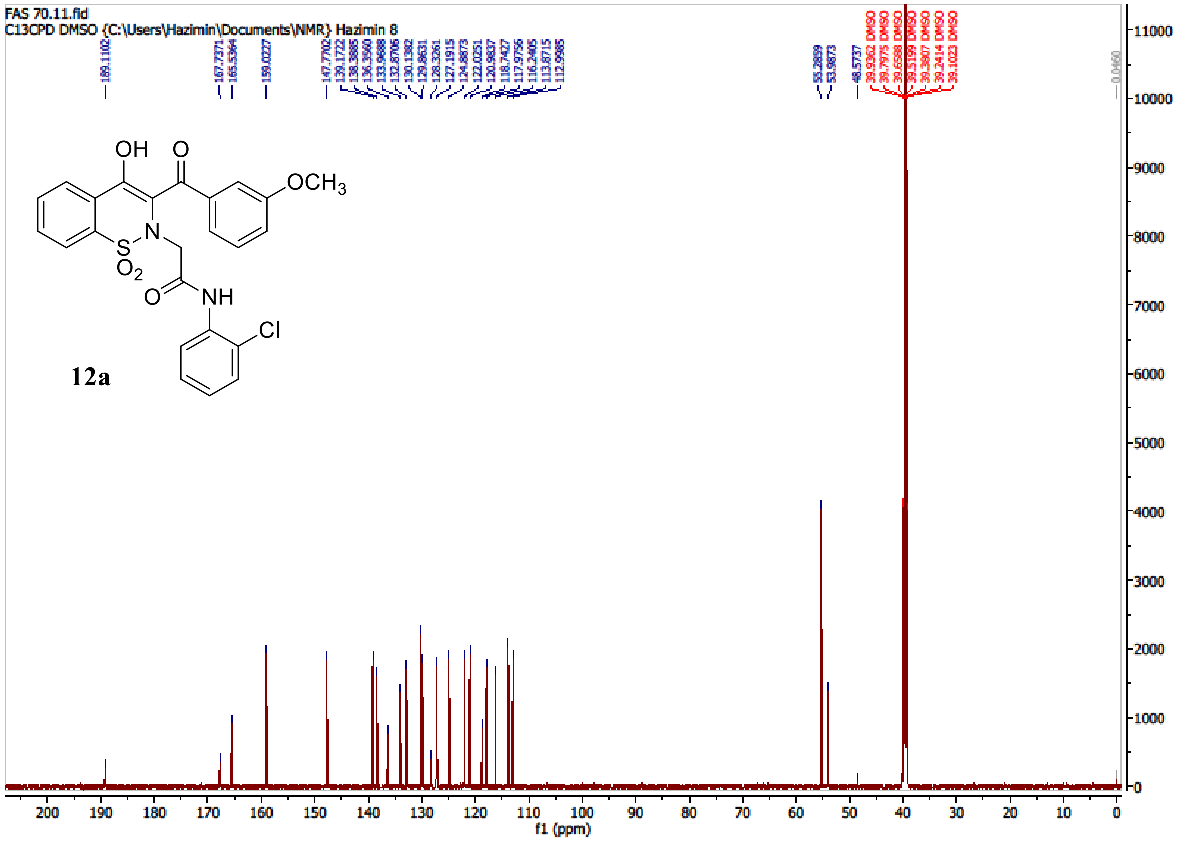

^{13}C NMR Spectrum of compound **12a.**

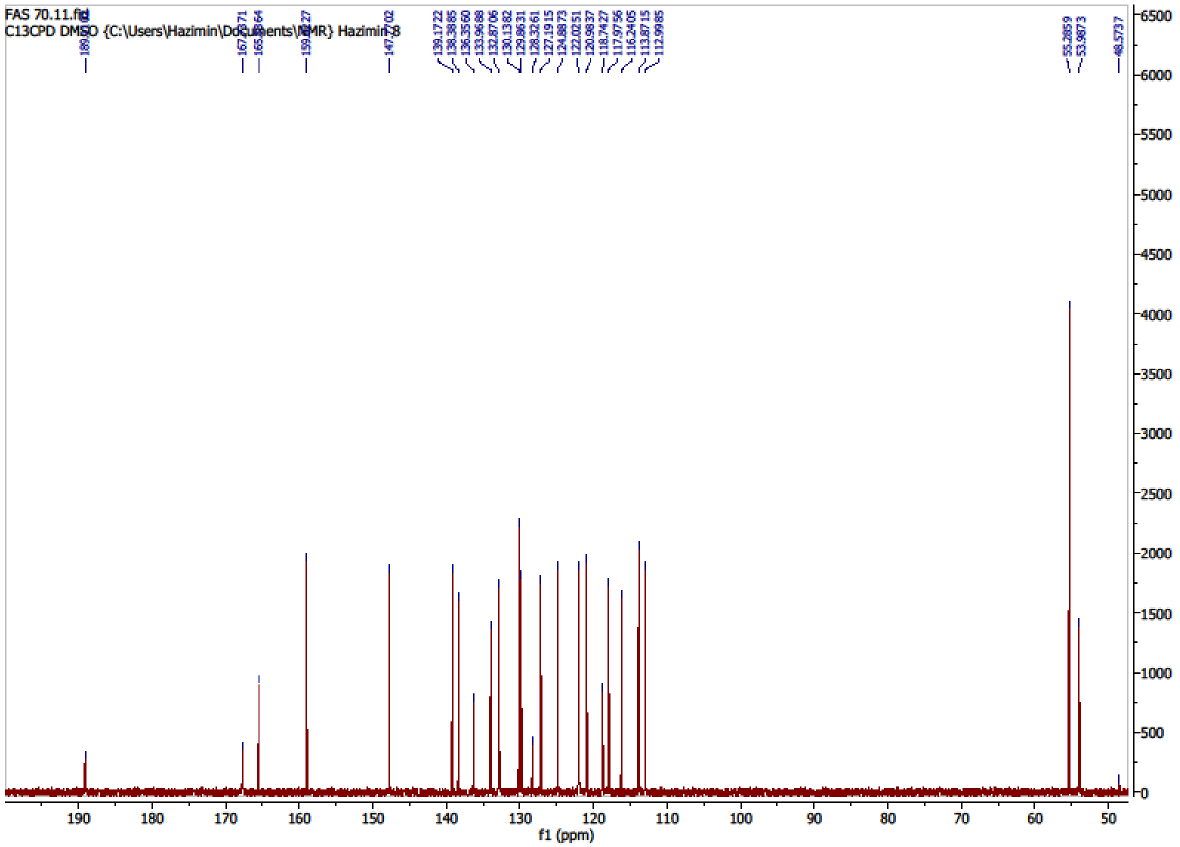

Expanded form of ^{13}C NMR Spectrum of compound **12a** (region 110-140 ppm).

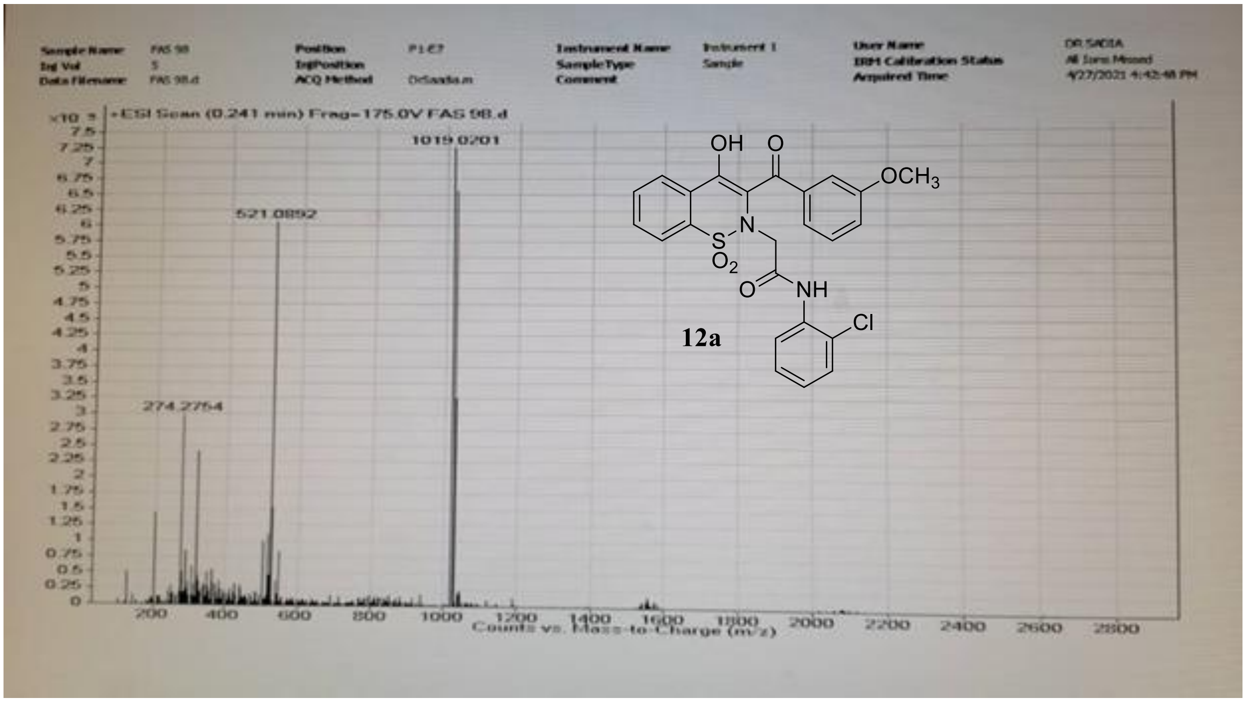

HRMS (ESI) Spectrum of compound **12a.**

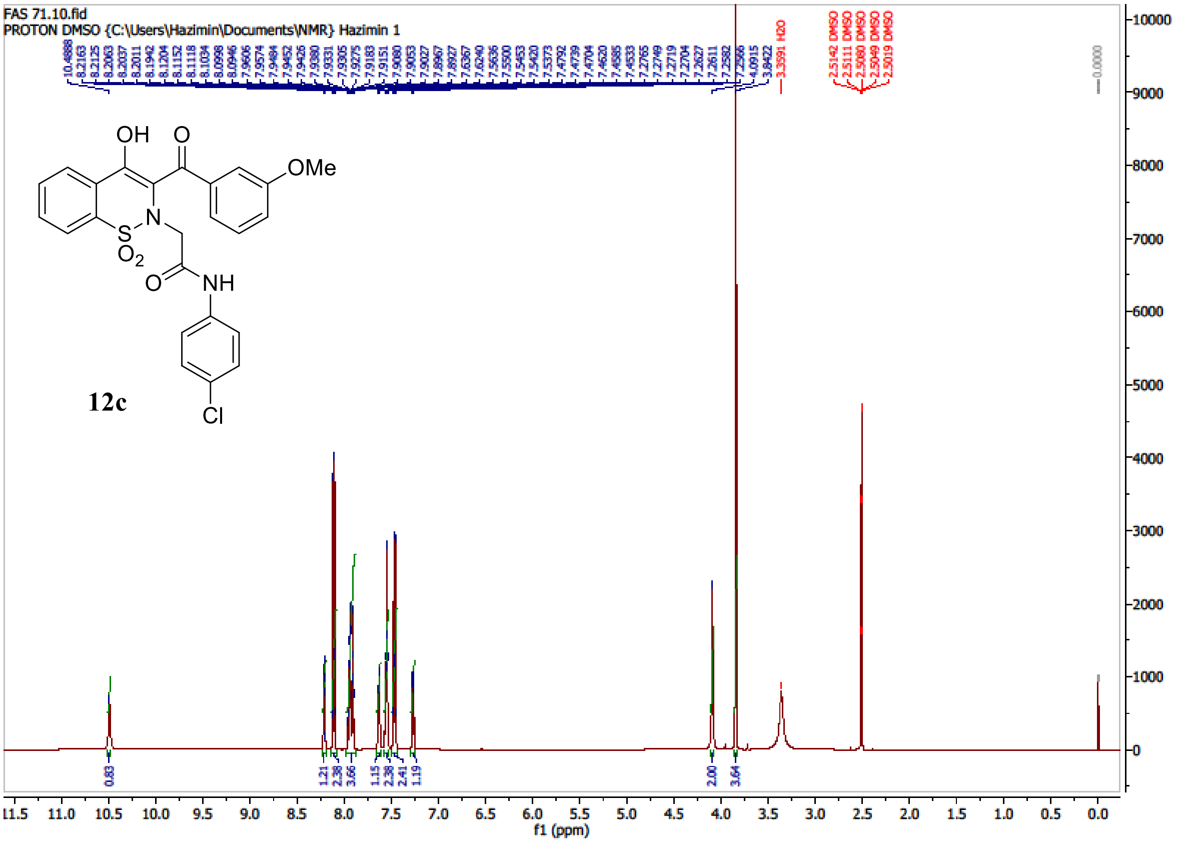

^{1}H NMR Spectrum of compound **12c**.

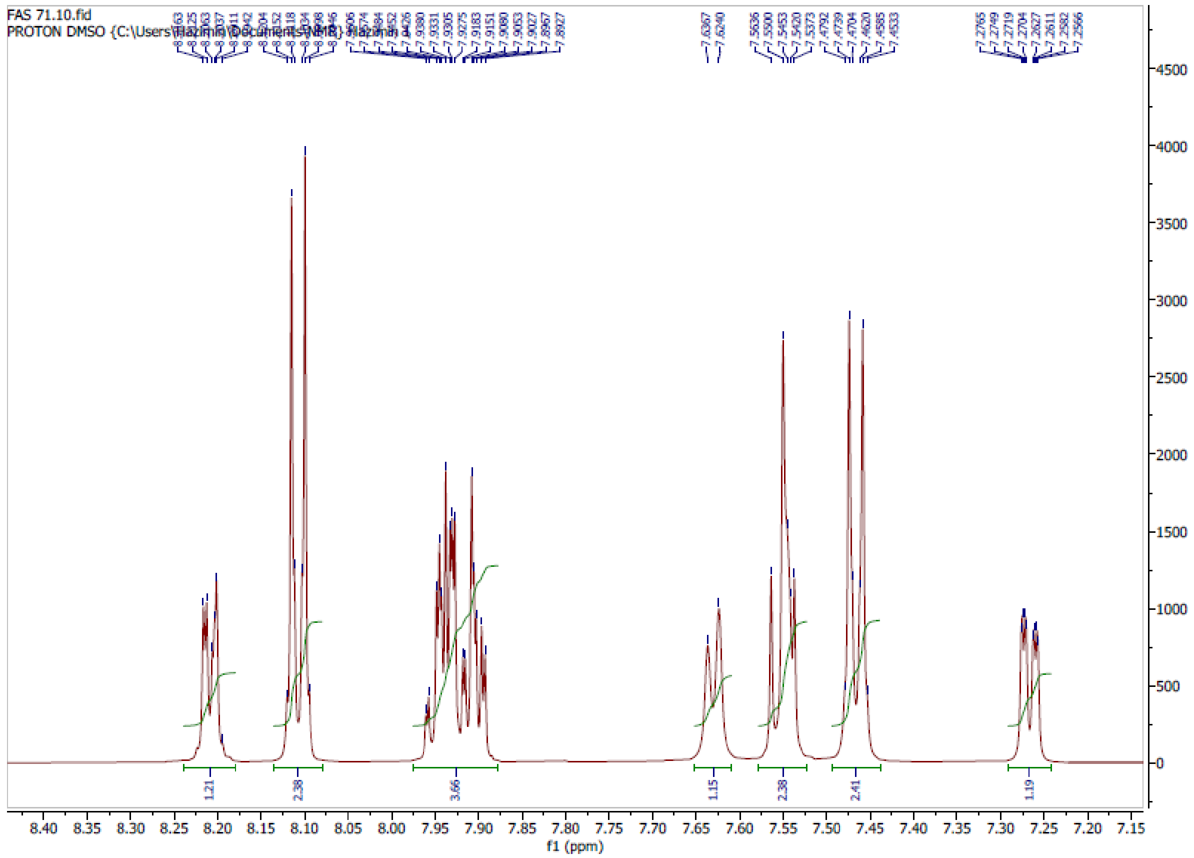

Expanded form of ^{1}H NMR Spectrum of compound **12c** (region 7.25-8.25 ppm).

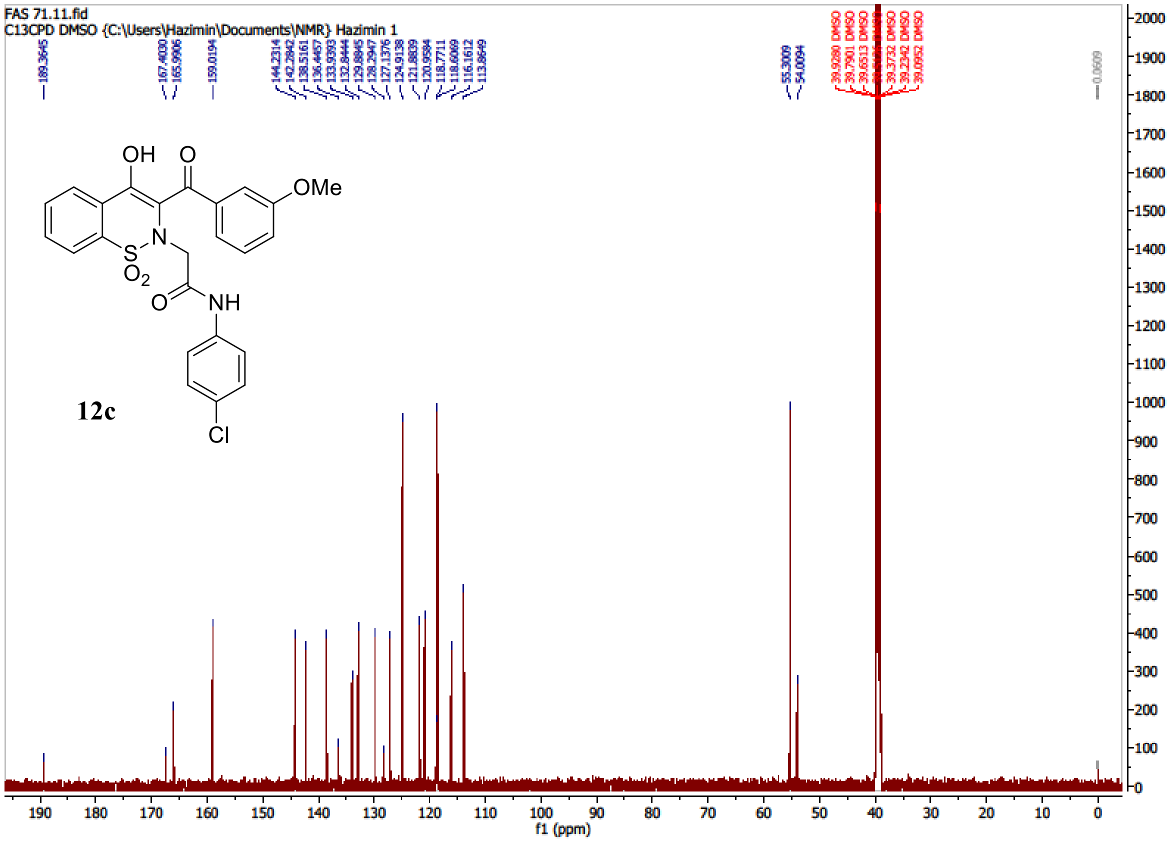

^{13}C NMR Spectrum of compound **12c.**

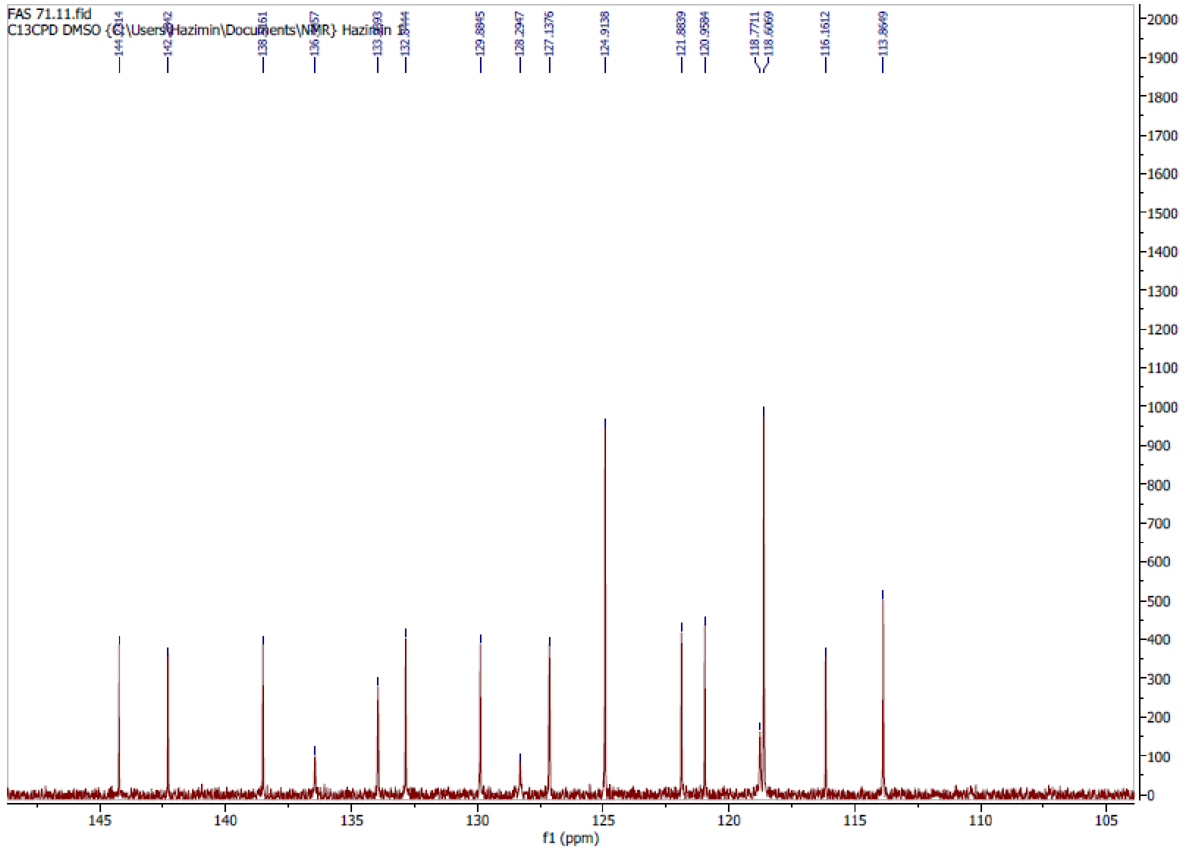

Expanded form of ^{13}C NMR Spectrum of compound **12c** (region 110-145 ppm).

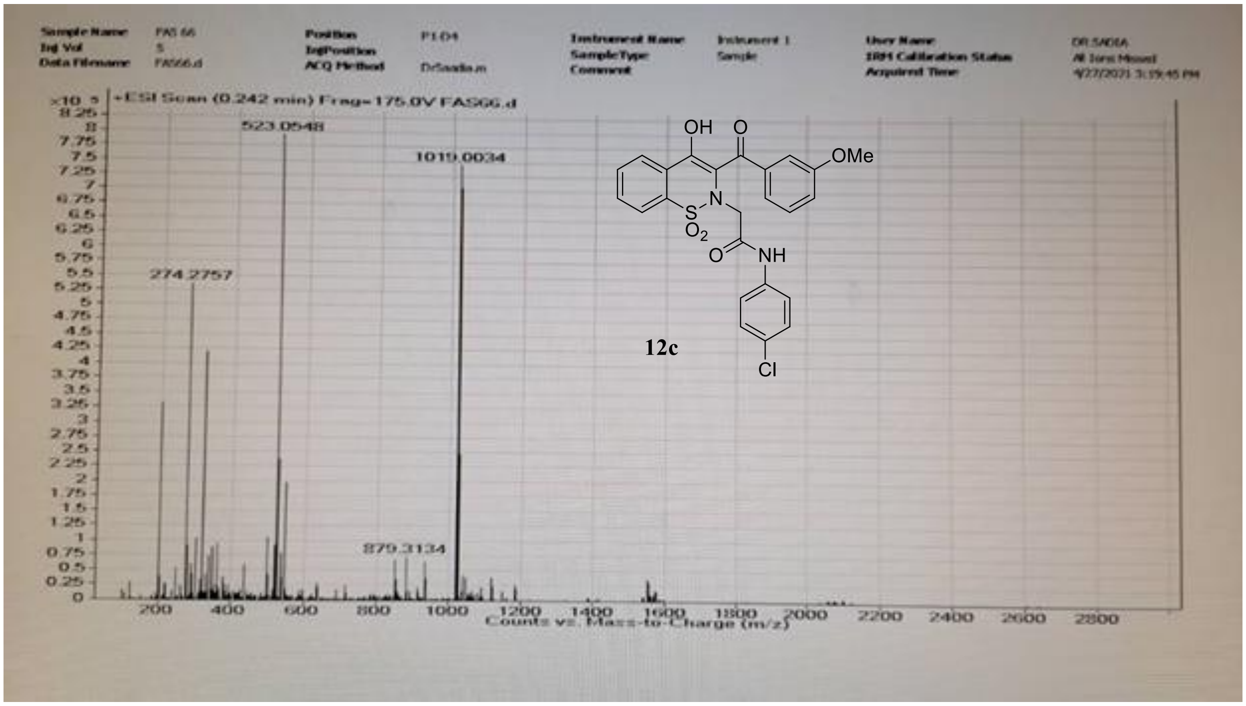

HRMS (ESI) Spectrum of compound **12c.**

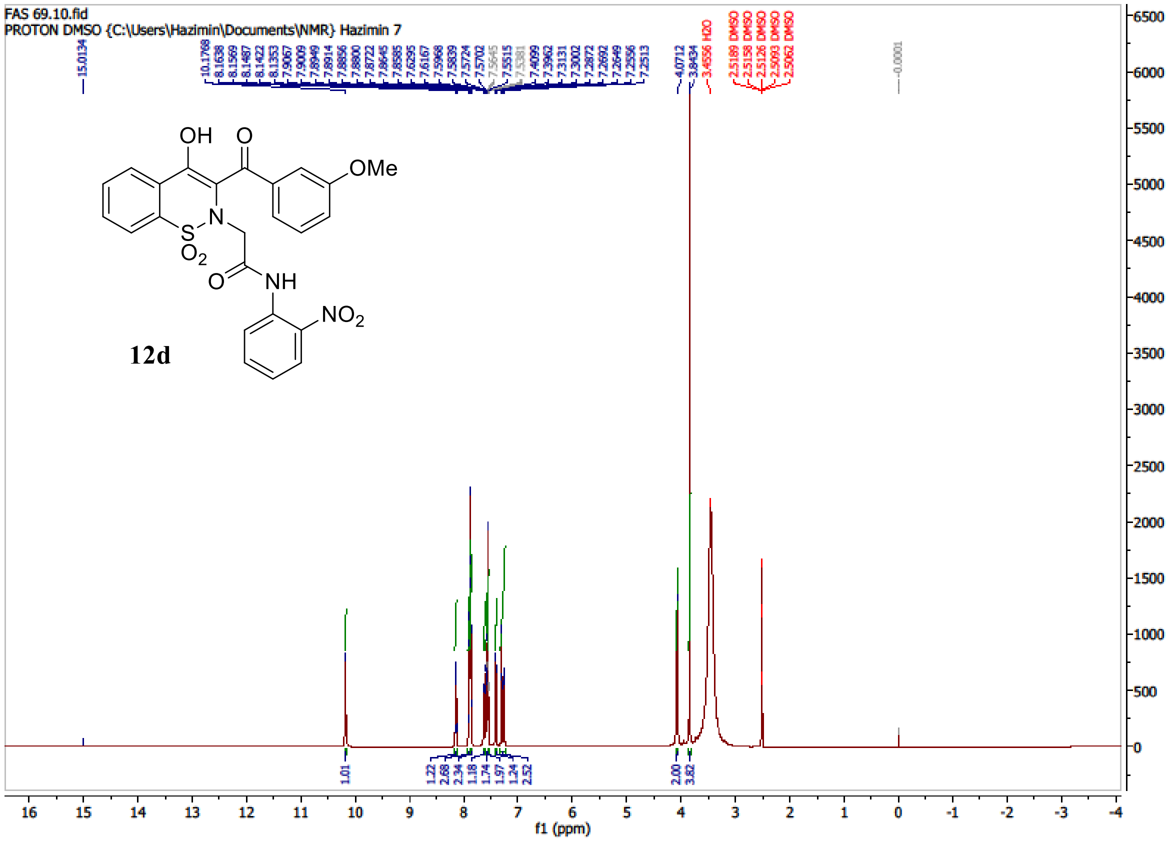

^{1}H NMR Spectrum of compound **12d**.

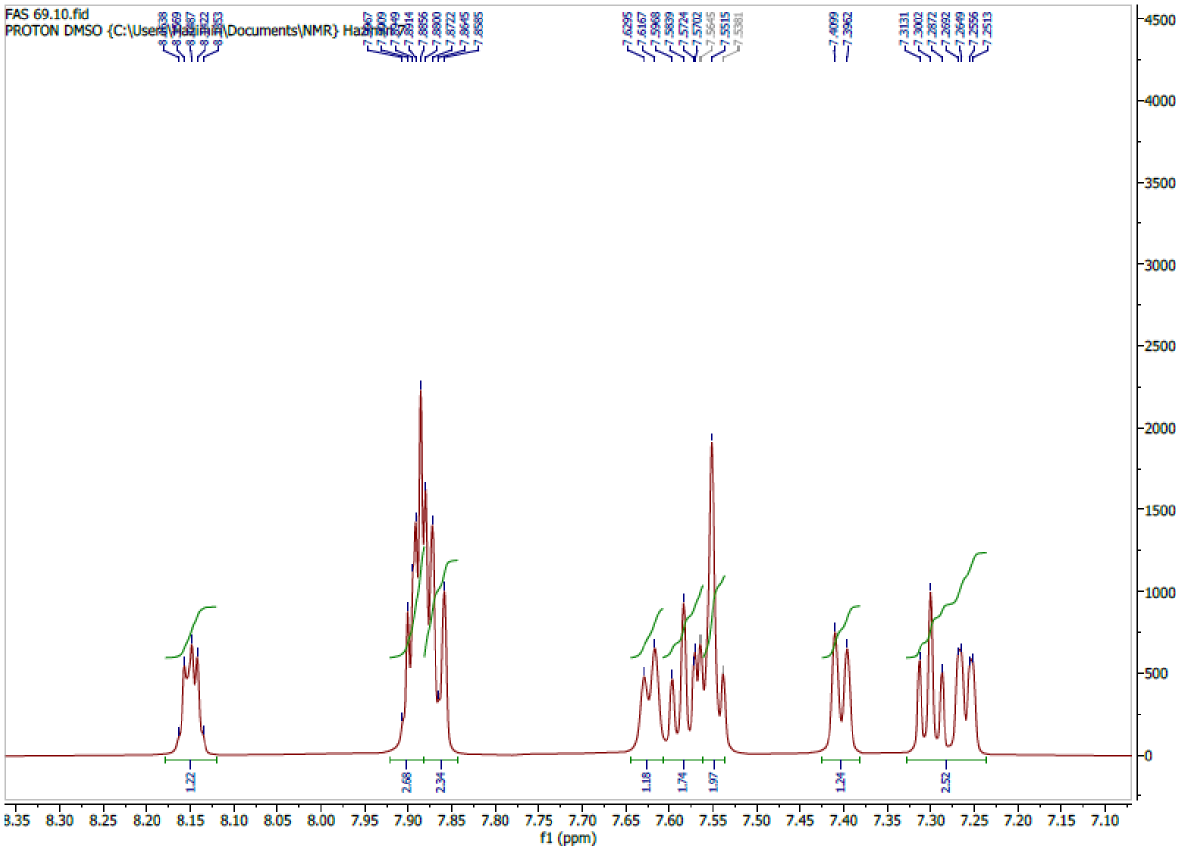

Expanded form of ^{1}H NMR Spectrum of compound **12d** (region 7.20-8.20 ppm).

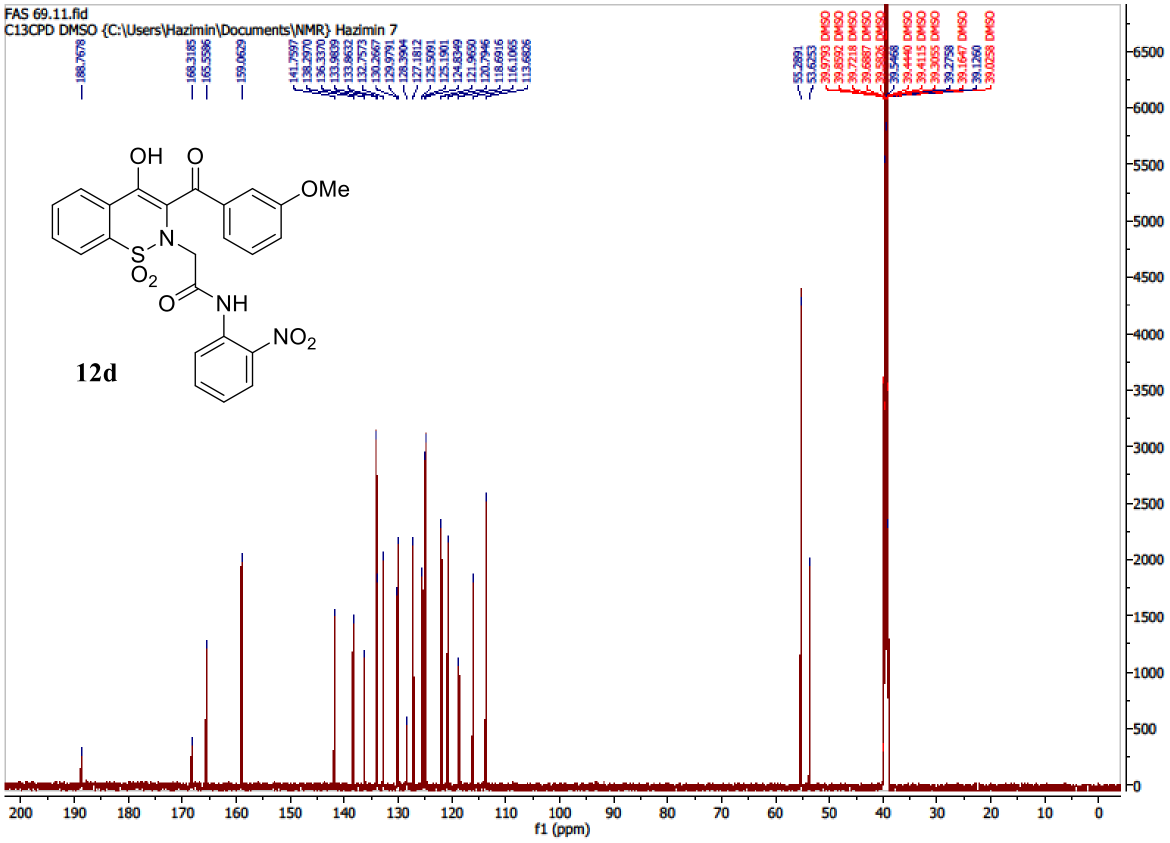

^{13}C NMR Spectrum of compound **12d.**

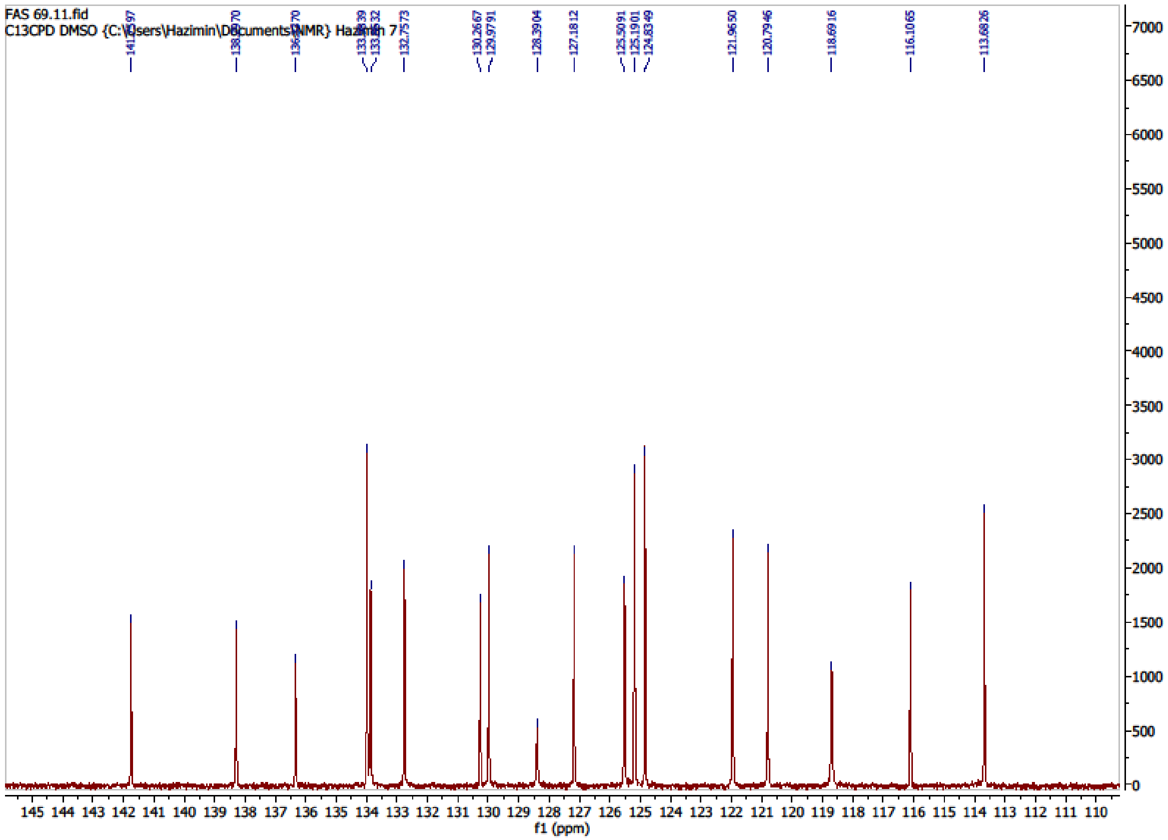

Expanded form of ^{13}C NMR Spectrum of compound **12d** (region 113-142 ppm).

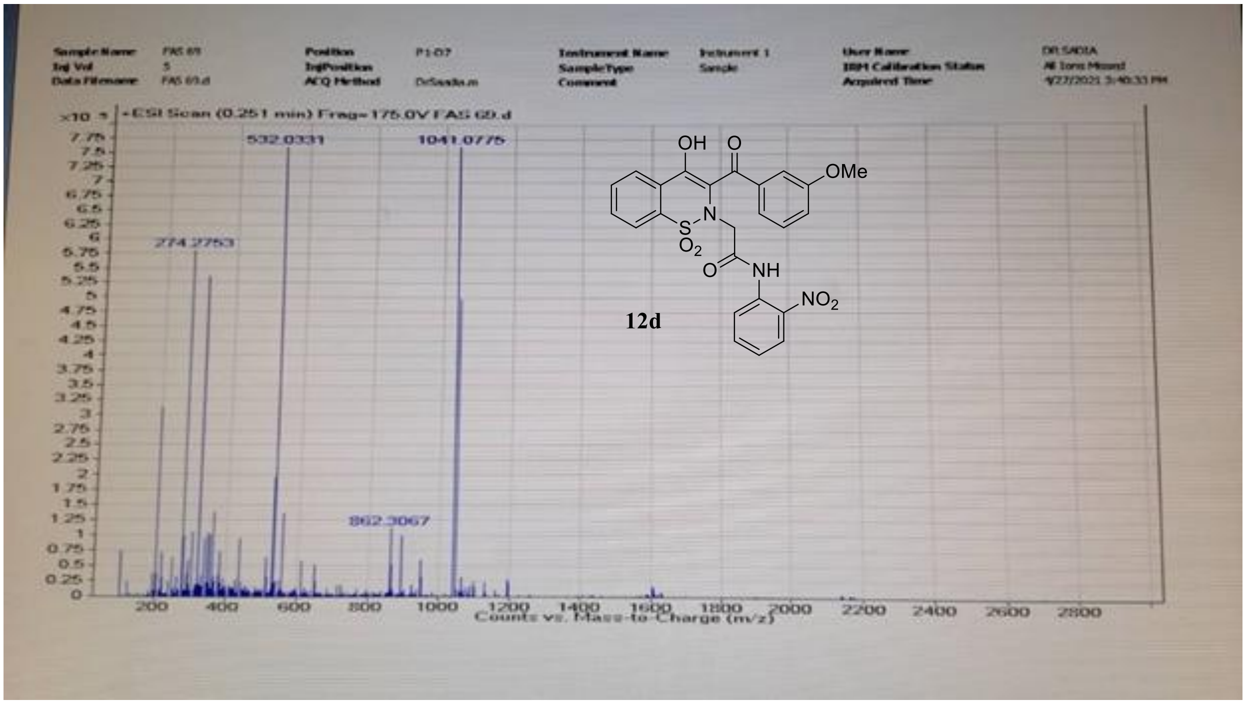

HRMS (ESI) Spectrum of compound **12d**.

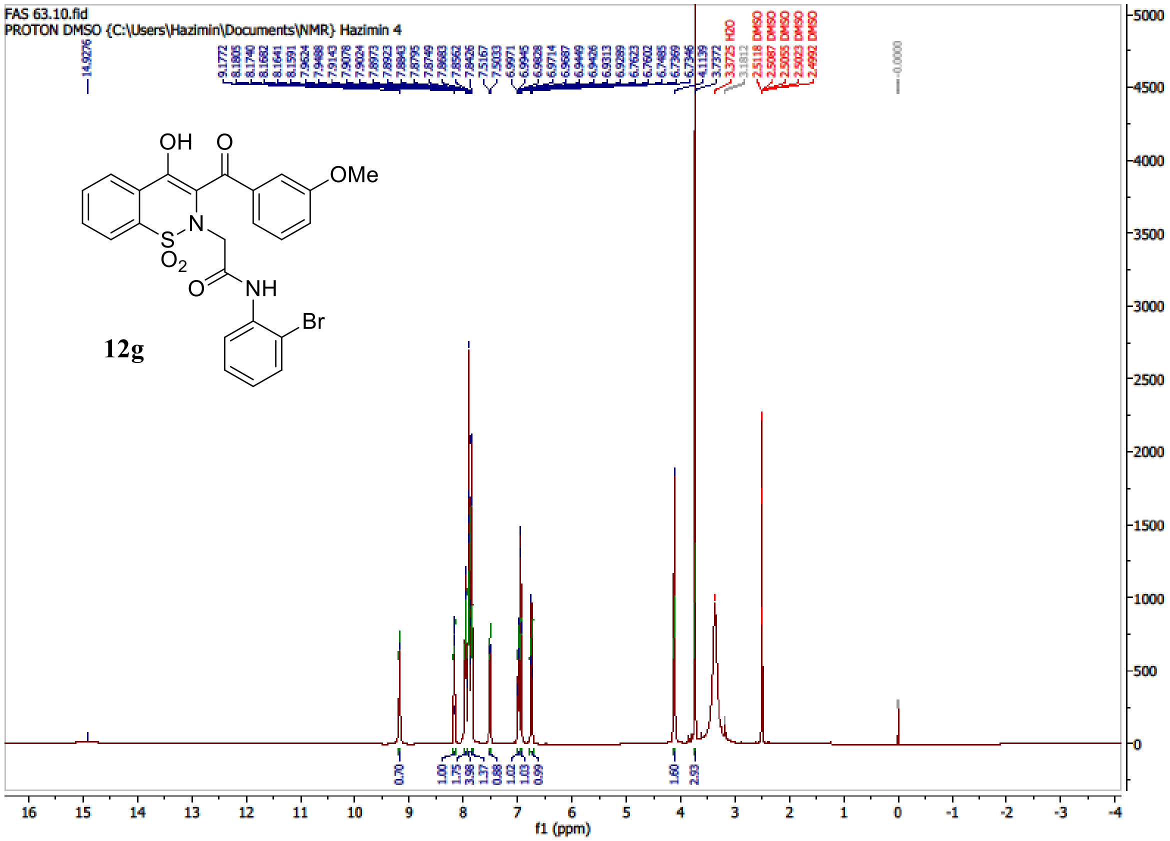

^{1}H NMR Spectrum of compound **12g**.

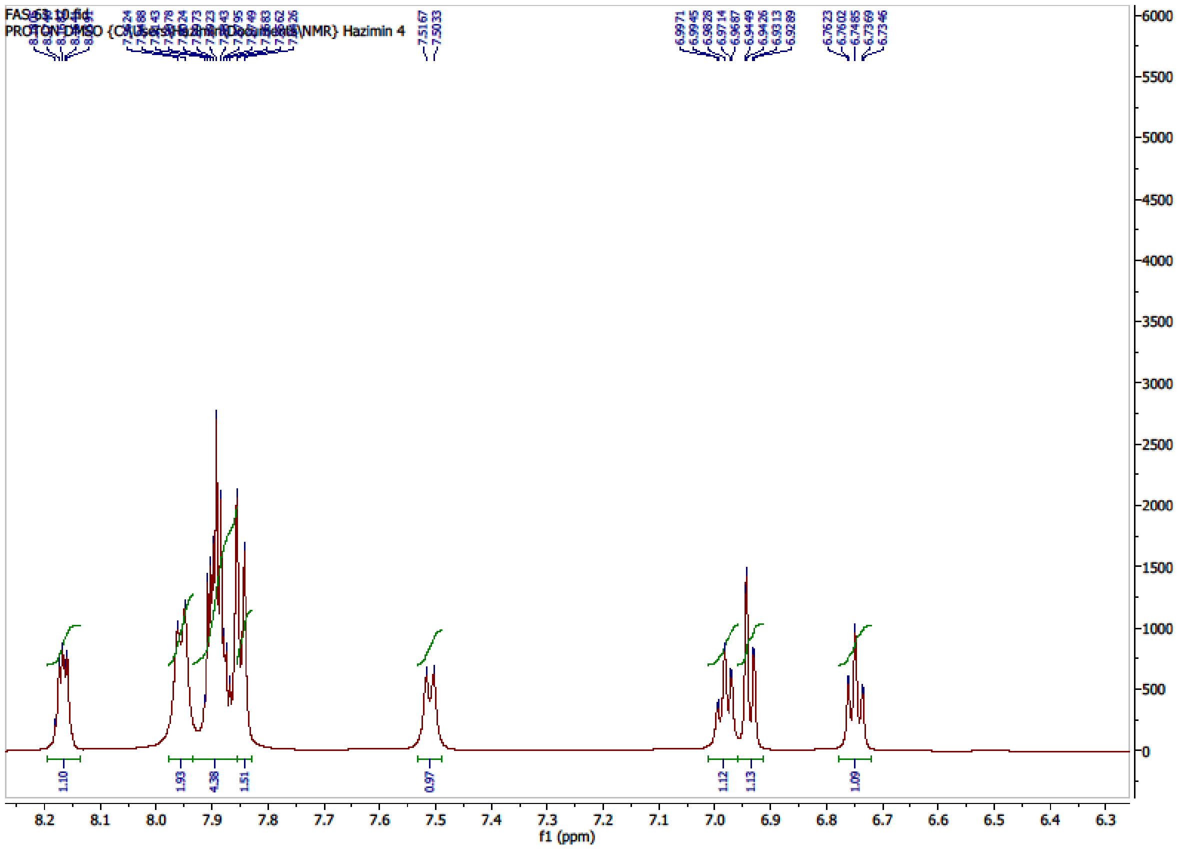

Expanded form of ^{1}H NMR Spectrum of compound **12g** (region 6.70-8.20 ppm).

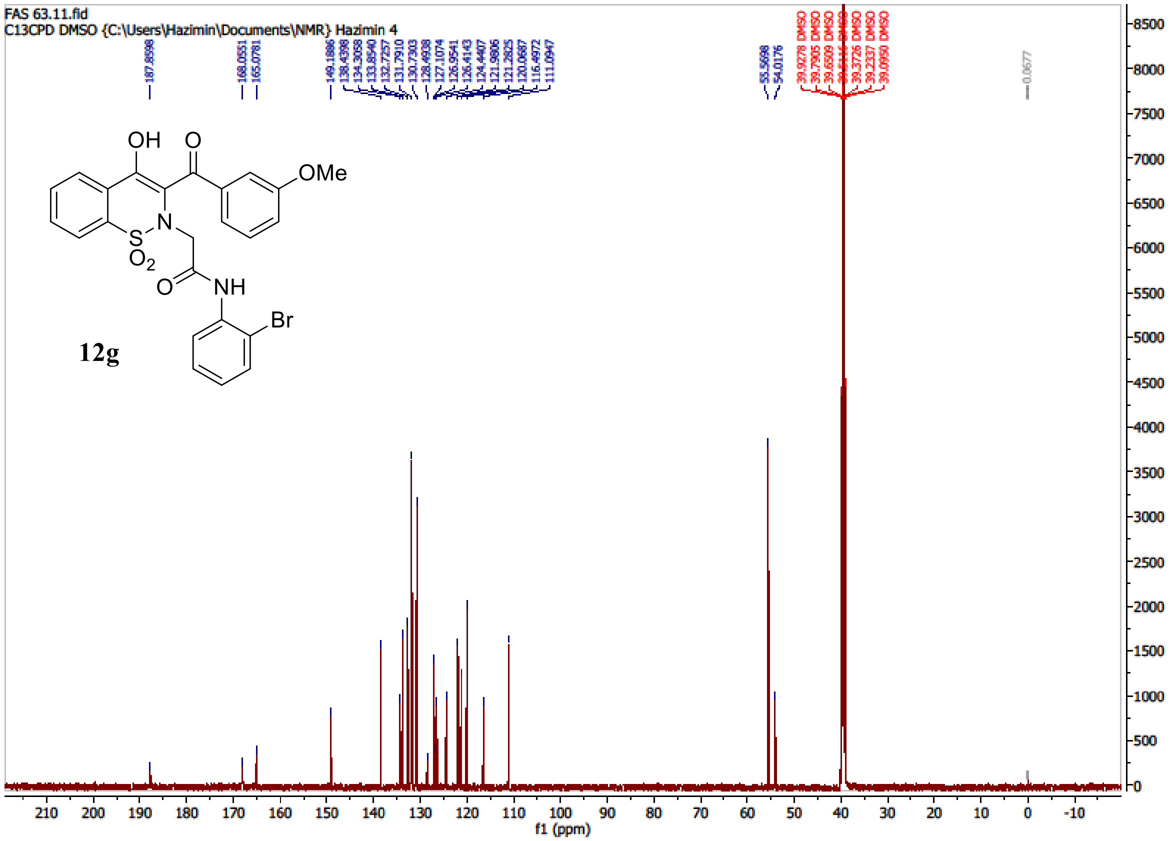

^{13}C NMR Spectrum of compound **12g.**

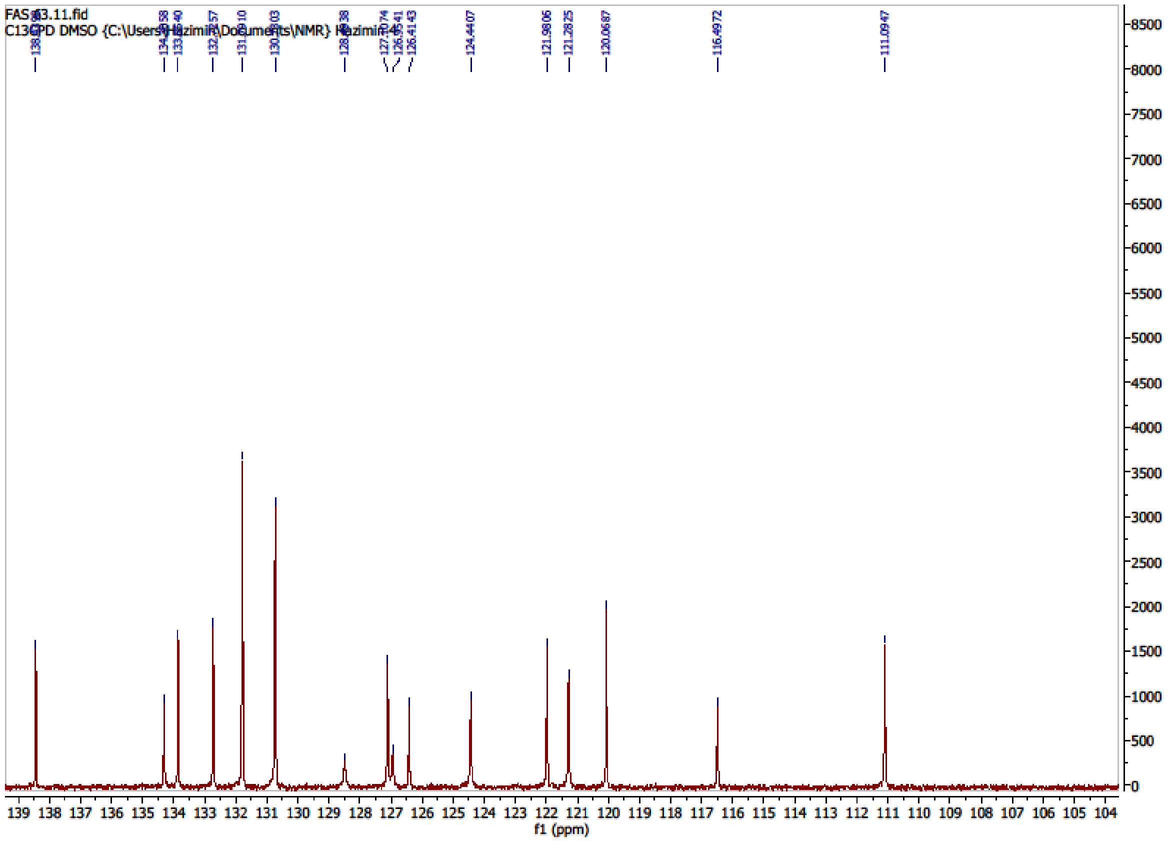

Expanded form of ^{13}C NMR Spectrum of compound **12g** (region 120-135 ppm).

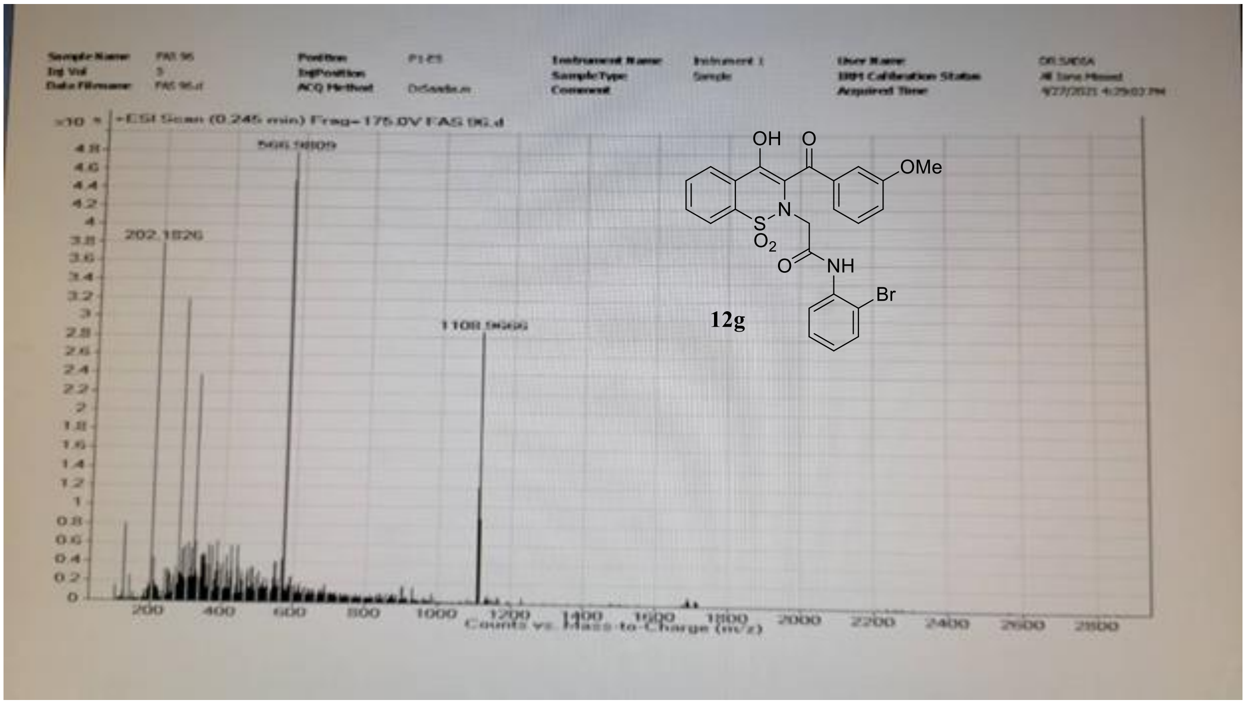

HRMS (ESI) Spectrum of compound **12g.**

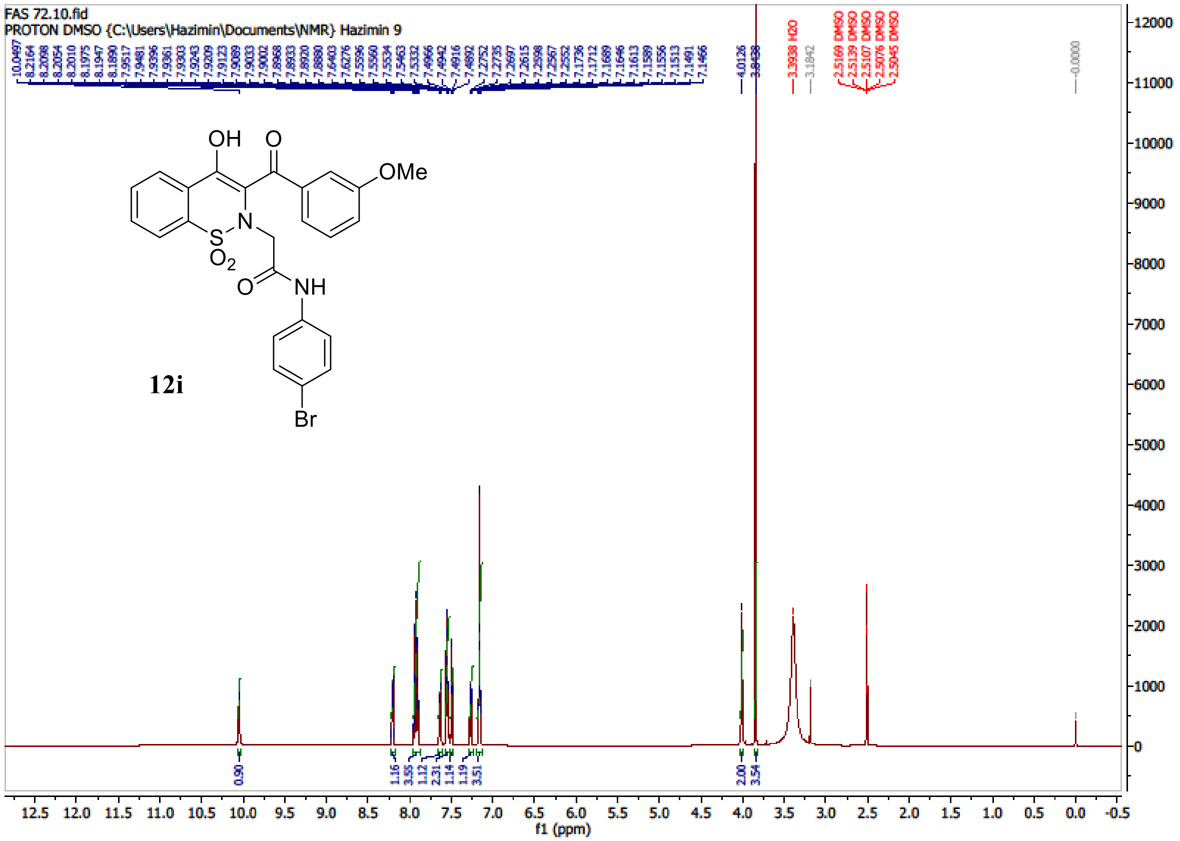

^{1}H NMR Spectrum of compound **12i**.

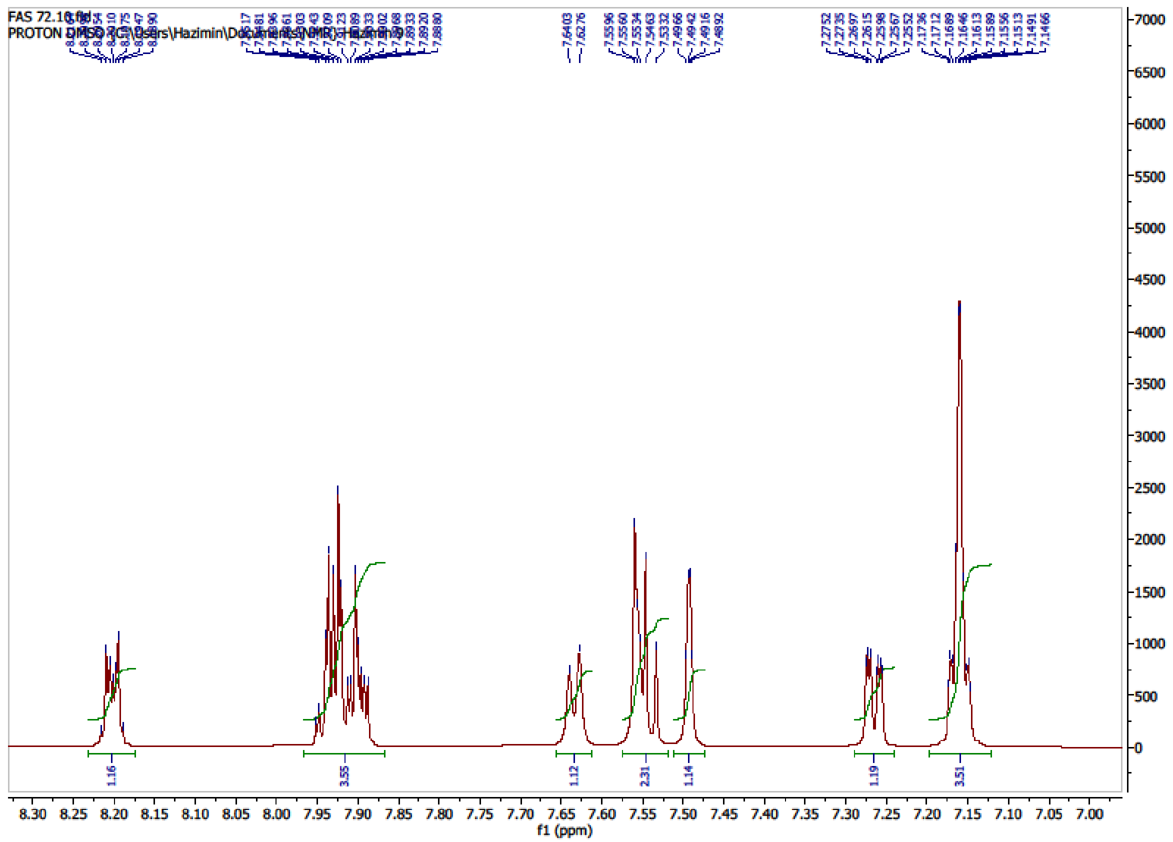

Expanded form of ^{1}H NMR Spectrum of compound **12i** (region 7.10-8.25 ppm).

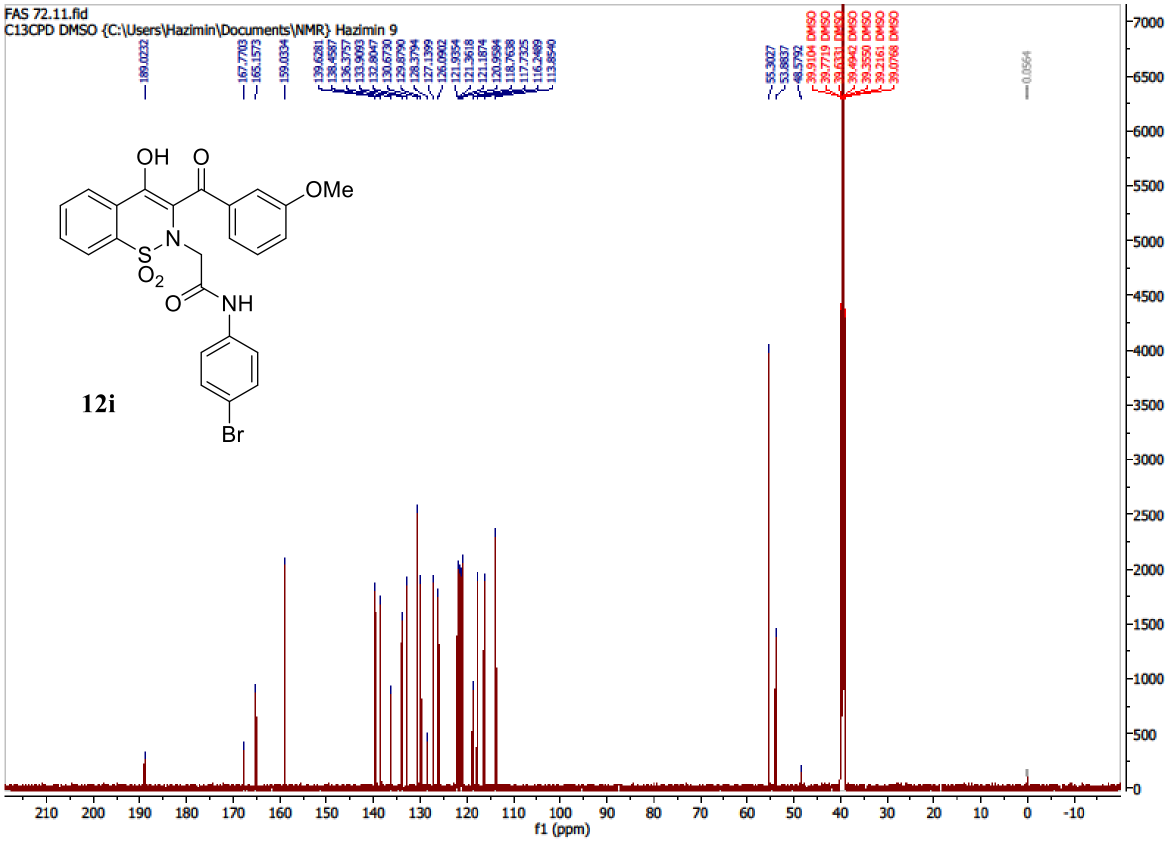

^{13}C NMR Spectrum of compound **12i.**

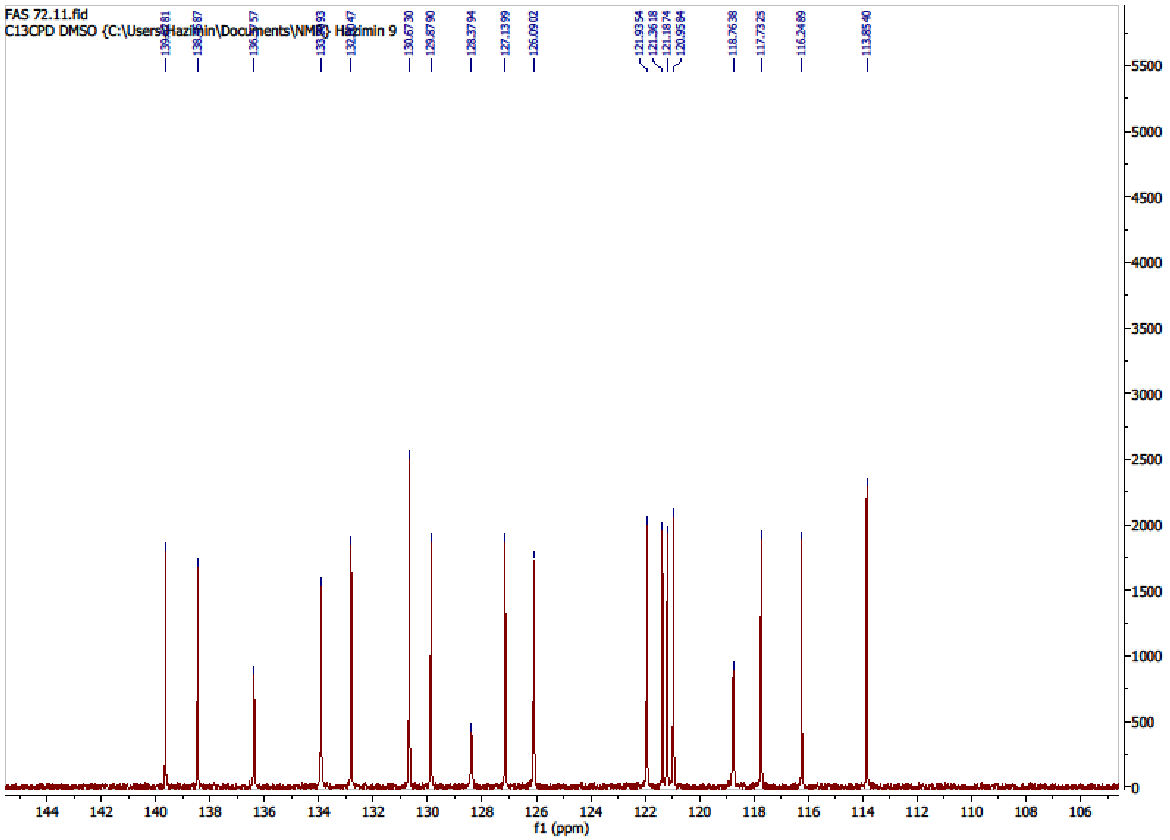

Expanded form of ^{13}C NMR Spectrum of compound **12i** (region 114-140 ppm).

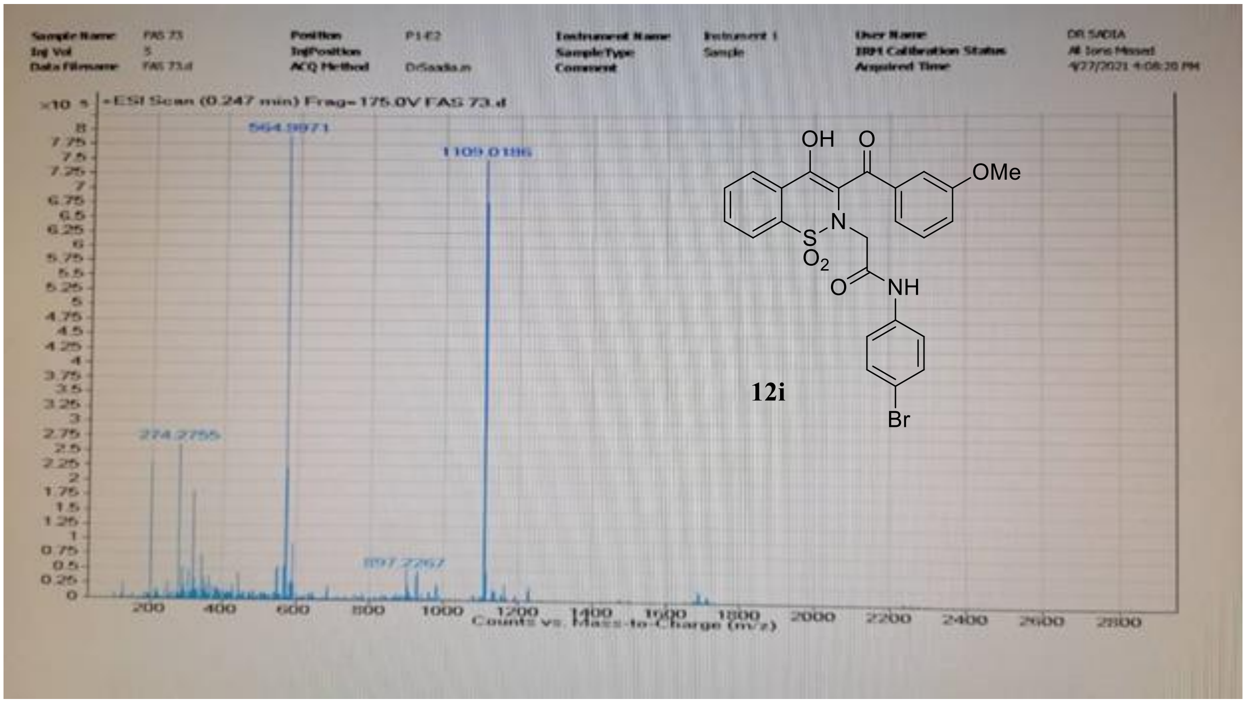

HRMS (ESI) Spectrum of compound **12i**.

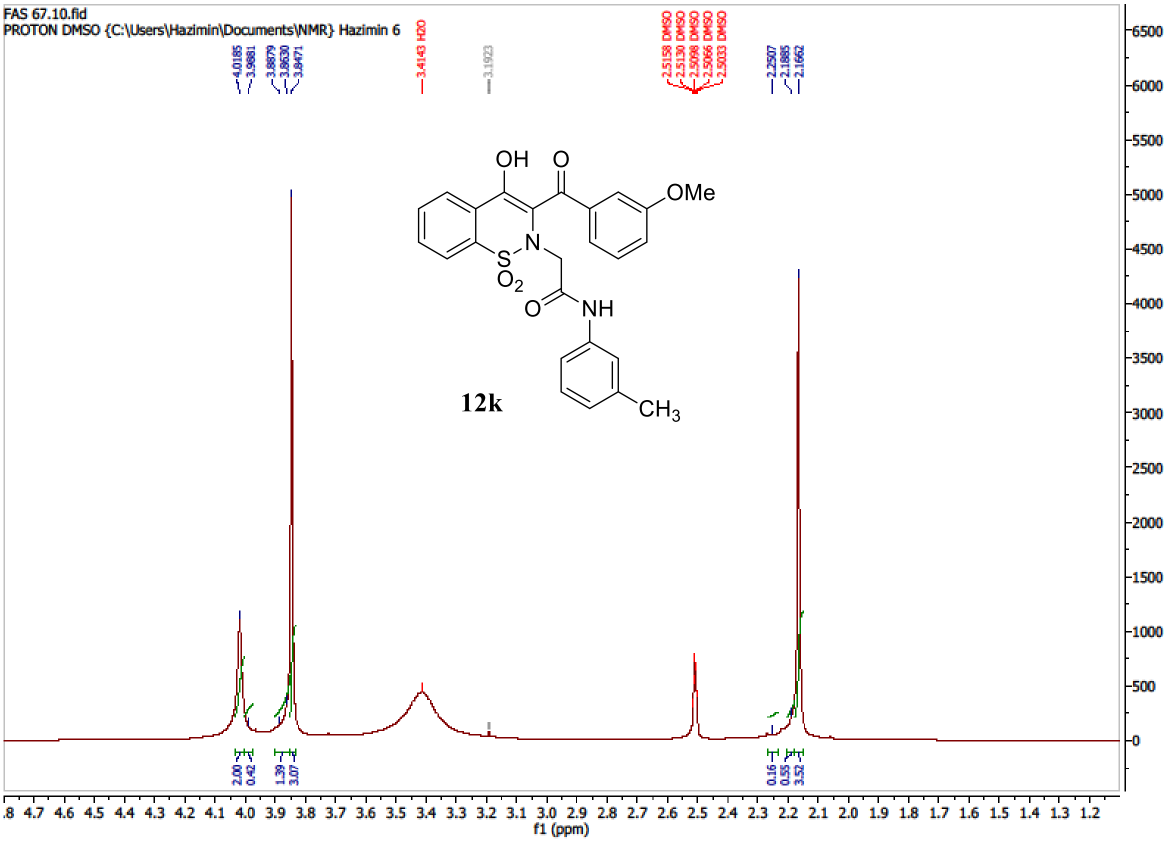

^{1}H NMR Spectrum (up-field region) of compound **12k**.

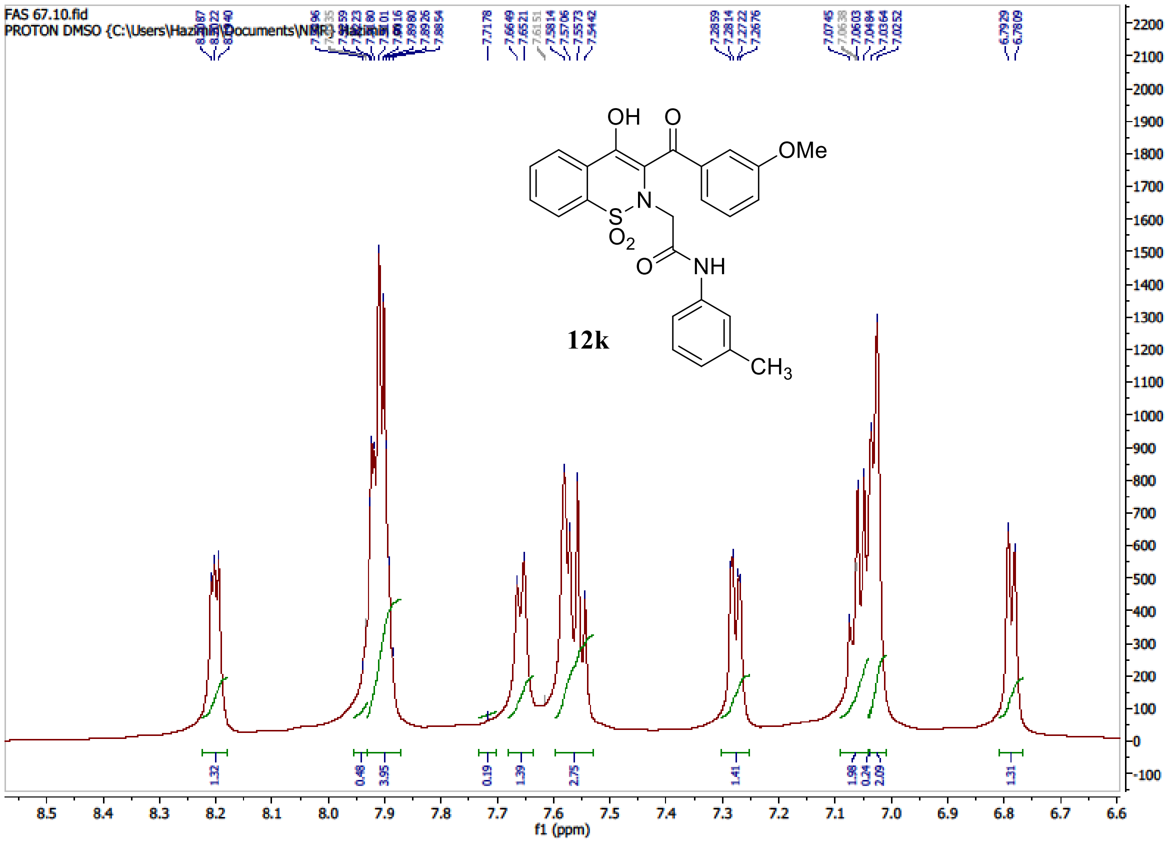

^{1}H NMR Spectrum (down-field region) of compound **12k**.

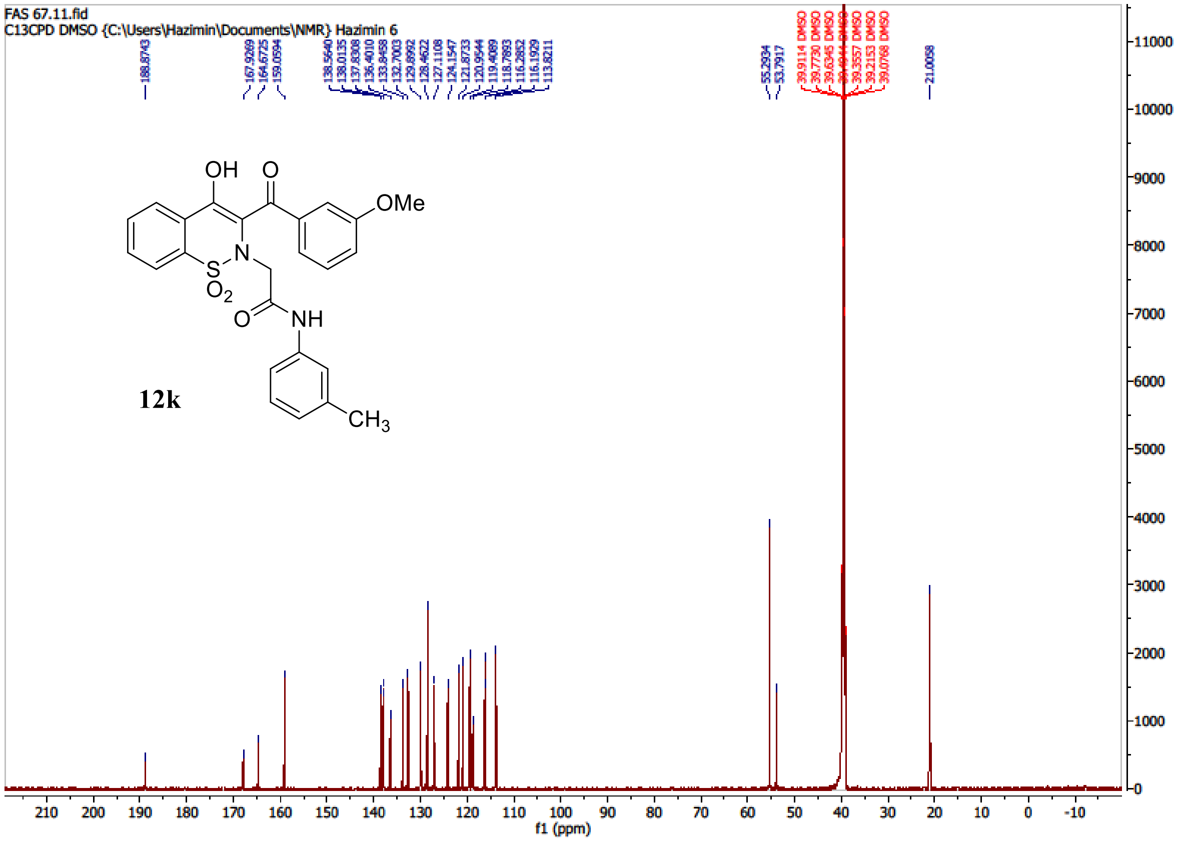

^{13}C NMR Spectrum of compound **12k.**

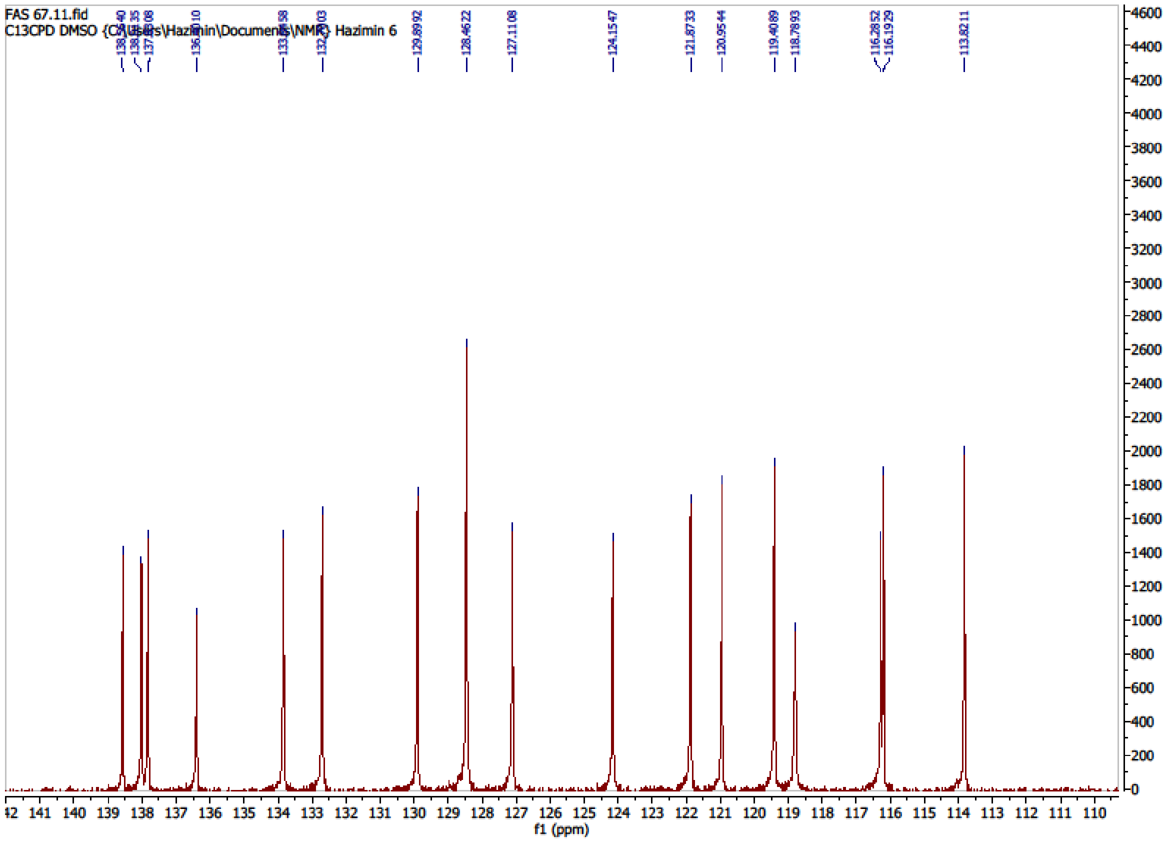

Expanded form of ^{13}C NMR Spectrum of compound **12k** (region 113-139 ppm).

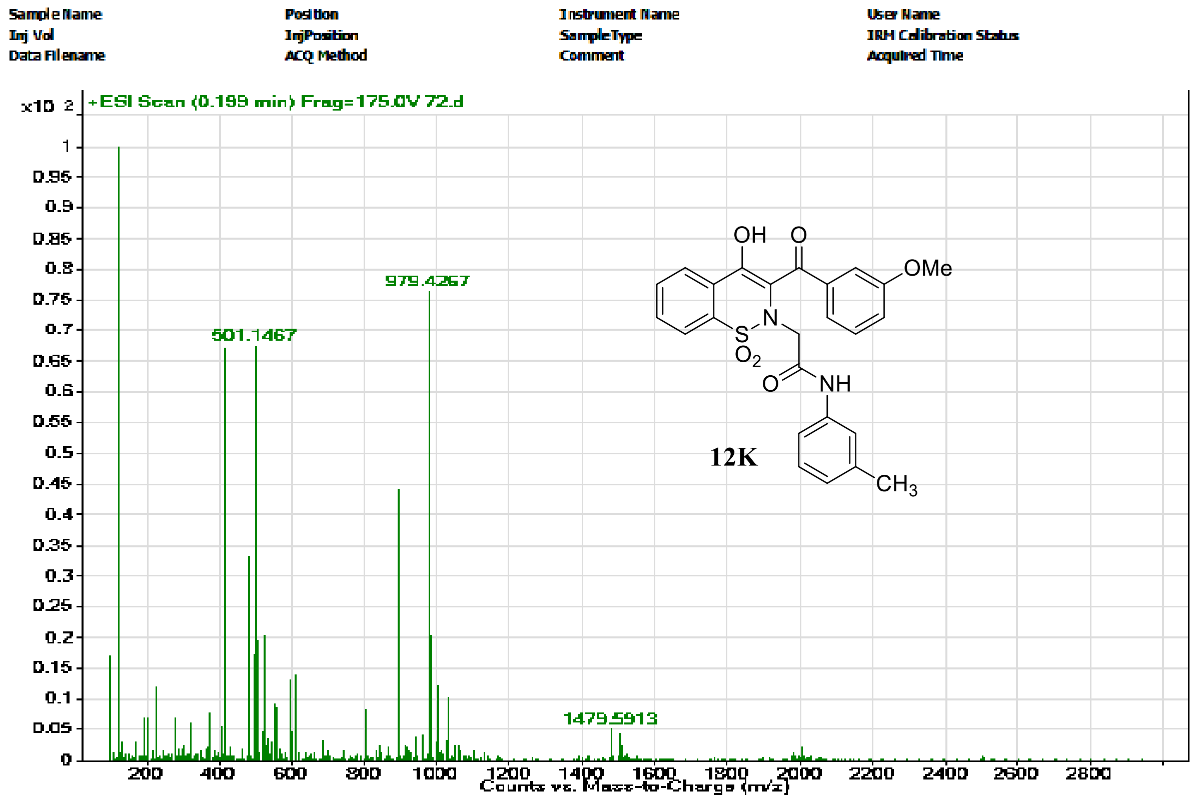

HRMS (ESI) Spectrum of compound **12k.**

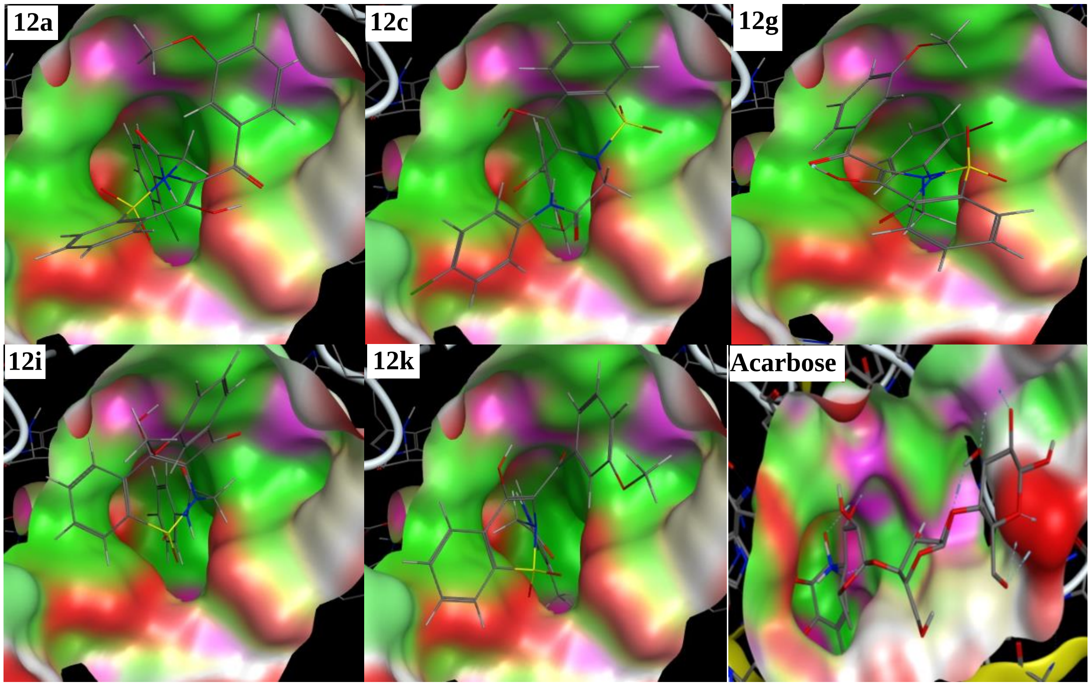

Figure S1. 3D docking modes of potent compounds (**12a, 12c, 12g, 12i, 12k**) and the reference drug, acarbose against α-glucosidase enzyme. Red color portions indicate the ligand exposure points and color portion shows pocket selected.

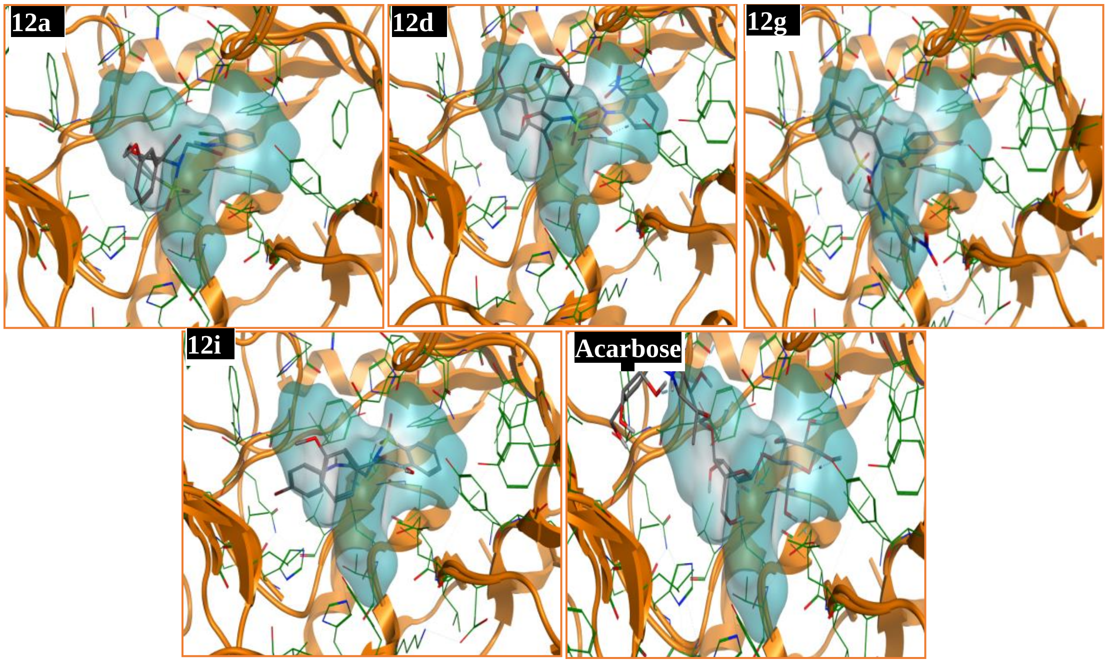

Figure S2. 3D docking modes of potent compounds (**12a, 12d, 12g, 12i**) and the reference drug, acarbose against amylase enzyme. Red color portions indicate the ligand exposure points and color portion shows pocket selected.
